# Application of Prodigiosin Extracts in Textile Dyeing and Novel Printing Processes for Halochromic and Antimicrobial Wound Dressings

**DOI:** 10.3390/biom15081113

**Published:** 2025-08-01

**Authors:** Cátia Alves, Pedro Soares-Castro, Rui D. V. Fernandes, Adriana Pereira, Rui Rodrigues, Ana Rita Fonseca, Nuno C. Santos, Andrea Zille

**Affiliations:** 1 Centre for Textile Science and Technology (2C2T), Department of Textile Engineering, University of Minho, Campus of Azurém, 4800-058 Guimarães, Portugal; catia.alves@2c2t.uminho.pt (C.A.); rdvfernandes@2c2t.uminho.pt (R.D.V.F.); adriana_a_pereira@hotmail.com (A.P.); ruipvrodrigues@hotmail.com (R.R.); 2 GIMM—Gulbenkian Institute for Molecular Medicine, Av. Prof. Egas Moniz, 1649-035 Lisbon, Portugal; pedromisc@gmail.com (P.S.-C.); ritafonseca-94@hotmail.com (A.R.F.); 3 Faculdade de Medicina, Universidade de Lisboa, Av. Prof. Egas Moniz, 1649-028 Lisbon, Portugal

**Keywords:** biopigment, *Pseudomonas*, sustainable textiles, novel printing, surfactants, biopolymers, dyeing process

## Abstract

The textile industry’s reliance on synthetic dyes contributes significantly to pollution, highlighting the need for sustainable alternatives like biopigments. This study investigates the production and application of the biopigment prodigiosin, which was produced by *Pseudomonas putida* with a yield of 1.85 g/L. Prodigiosin was prepared under acidic, neutral, and alkaline conditions, resulting in varying protonation states that influenced its affinity for cotton and polyester fibers. Three surfactants (anionic, cationic, non-ionic) were tested, with non-ionic Tween 80 yielding a promising color strength (above 4) and fastness results with neutral prodigiosin at 1.3 g/L. Cotton and polyester demonstrated good washing (color difference up to 14 for cotton, 5 for polyester) and light fastness (up to 15 for cotton, 16 for polyester). Cellulose acetate, used in the conventional printing process as a thickener, produced superior color properties compared to commercial thickeners. Neutral prodigiosin achieved higher color strength, and cotton fabrics displayed halochromic properties, distinguishing them from polyester, which showed excellent fastness. Prodigiosin-printed samples also exhibited strong antimicrobial activity against *Pseudomonas aeruginosa* and retained halochromic properties over 10 pH cycles. These findings suggest prodigiosin as a sustainable dye alternative and pH sensor, with potential applications in biomedical materials, such as antimicrobial and pH-responsive wound dressings.

## 1. Introduction

The textile industry is one of the most polluting industries worldwide, mainly due to the large use of harmful chemical compounds. Synthetic dyes are one of the major textile pollutants, with an annual production of 700,000 tons of over 100,000 different dyes [1]. After dyeing and finishing processes, up to 50% of dyestuff can reach nearby bodies of water, contaminating the environment [2]. The substitution of synthetic dyes for greener alternatives is crucial for the transition to a sustainable, circular textile industry and for future generations, fighting climate change and preserving natural ecosystems. Greener textile processes will reduce water consumption, carbon dioxide emissions, and the release of hazardous wastewaters to the environment [2,3]. In recent years, researchers have been focused on substituting synthetic dyes for eco-friendlier fabric coloration methods such as biodyes and biopigments originating from plants [4,5,6,7,8], structural coloration [9,10], and biopigments produced by a wide range of microorganisms as secondary metabolites, including bacteria, fungi, and algae [11,12,13,14]. Most recently, Carvalho et al. compared the functionalization of different fabrics with lycopene extracts (from tomato and watermelon). Under specific dyeing conditions, watermelon extracts produced coloration in polyester fabrics comparable to that achieved using a commercial disperse dye [15].

In particular, the versatility and sustainability of microbial processes and products have driven research efforts toward establishing large-scale fermentation and textile application of microbial biopigments, because, in contrast to plant-derived compounds, their production is not limited by climate, season, or region [16]. Microorganisms are easy to cultivate, being able to achieve fast growth rates, supported by the use of inexpensive substrates, which in many cases may be waste products or byproducts of other industrial processes, and can be genetically and metabolically engineered to boost native or heterologous biosynthetic pathways of virtually any biopigment [11,17]. Nevertheless, several aspects of the microbial production of biopigments still require knowledge-based improvement in order to establish biotechnological processes as their leading source. These aspects include sub-optimal fermentation yields, non-optimal production, higher costs when compared to the utilization of synthetic dyes, and challenges in biopigment recovery steps downstream of the fermentation stage, among others [11,18]. Moreover, the implementation of biopigments in the textile industry can be cost-effective (raw materials, growth media, and substrates) as biopigment production requires specialized and expensive equipment, being time-consuming [19]. However, bacterial pigments represent non-toxic, sustainable colorants that support vibrant textile dyeing and low-impact production, free from ethical concerns [20].

Several microbial-derived biopigments, including violacein, melanin, indigo, and carotenoids, have already been characterized for their promising properties with biotechnological relevance (e.g., organoleptic properties, inhibiting oxidation or generating free radicals, suppressing microbial growth, absorbing ultraviolet (UV) radiation) [17]. These bioactive pigments not only offer a wide range of colors but may also confer relevant functionalities to dyed textiles, such as antimicrobial, antioxidant, and UV-protective properties, thereby adding value and versatility to textile materials [11].

**Figure 1:**
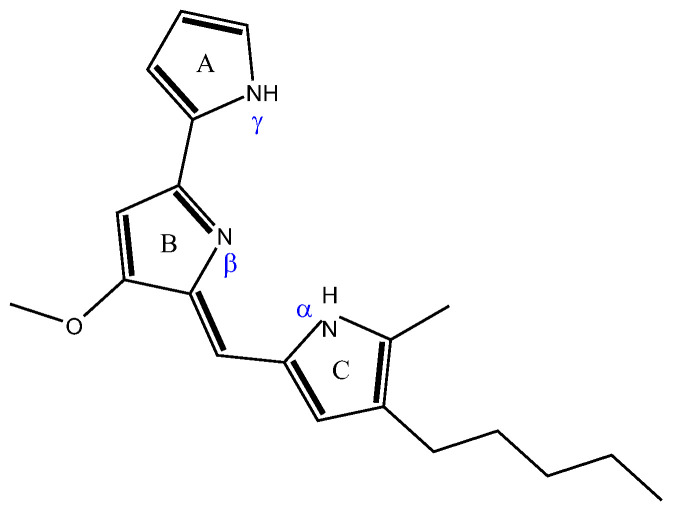
Prodigiosin chemical structure.

Prodigiosin is a red biopigment (Figure 1) natively produced by several bacterial species, commonly studied in members of the genus *Serratia*, whose prodigiosin production yield has been reported to range from 0.09 to 50 g/L [21,22]. However, *Serratia* spp. are potential human and animal pathogens, and thereby, the heterologous production of prodigiosin has also been exploited in other bacterial strains. For example, Pseudomonas putida KT2440-derived strains PIG02 and pig21 are compatible with industrial applications, exhibiting reduced pathogenicity compared to *Serratia* spp. [22,23,24,25,26]. *P. putida* strains, and in particular strain KT2440, from which strain PIG02 was derived, are robust and safe hosts for heterologous production of bioactive compounds like prodigiosin. Unlike native producers such as *Serratia* spp., which are potential human pathogens, *P. putida* is HV1-certified, making it suitable for industrial and laboratory use without biosafety concerns [23]. Its metabolism has been extensively characterized, offering a strong foundation for metabolic engineering, including the integration of complex biosynthetic pathways [24,25]. Strain’s metabolic versatility allows it to utilize a wide range of carbon and nitrogen sources, maintain high Adenine Dinucleotide Phosphate regeneration rates, and tolerate redox-intensive processes, such as the biosynthesis of redox pigments like prodigiosin [24]. Therefore, with a well-established molecular toolbox for metabolic engineering, scalability from lab to industry, and validated capability to produce prodigiosin, *P. putida* is a reliable bacterial strain for sustainable, safe, and efficient prodigiosin biosynthesis. Nonetheless, the yields of recovered prodigiosin reported for *P. putida* strains reached values of up to 1.10 g/L, considerably lower than the maximum yields observed in some studies with *Serratia* spp. An improvement in prodigiosin yield from heterologous production may be achieved through further molecular and physiological optimizations.

Prodigiosin contains three pyrrole units (Figure 1), with protonation state-dependent absorption spectral shifts, leading to different pigment colors depending on the pH conditions. Typically, solutions of prodigiosin extracted from *Serratia* spp. are halochromic, as they are pink at acidic pH, red at neutral pH, and yellow at alkaline pH [22,27].

Prodigiosin has several biomedical activities (antibacterial, anticancer, immunosuppressive, among others) and has also been previously studied for its dyeing potential in different types of fibers [21,27,28,29,30,31,32,33,34]. Asitok et al. studied the influence of varying washing conditions on the observed color after dyeing cotton (CO), wool (WO), and silk (SK) with prodigiosin extracted from *Serratia marcescens* [33]. Overall, the fabric that retained the most pigment was WO, followed by CO and SK. It was noticed that color intensity decreased when WO was washed with an acidic solution and increased when it was washed with an alkaline solution. The fact that one type of fiber retained more pigment is indicative that the chemical composition of the fiber plays a crucial role in its affinity to prodigiosin. This was also observed in the studies of Metwally et al. and Kramar et al., where it was noticed that synthetic fibers, such as polyester (PES) and polyamide (PA), had a more intense color (due to higher affinity) than cellulosic fibers [27,30]. This affinity is dependent on the ability of the fabric to make hydrogen bonds with prodigiosin. For example, in PES fabric, the oxygen atoms from the ester group can form more hydrogen bonds with pyrrole units than the -OH groups in cellulosic fabrics. Furthermore, the prodigiosin protonation state also influences the formation of these bonds, with neutral prodigiosin forming more hydrogen bonds than positively or negatively charged prodigiosin molecules [35]. Metwally et al. also took advantage of the hydrogen bonding ability of prodigiosin to produce copper and iron complexes to change the color and intensity, according to the metal ion in the complex [30]. Zhang et al. prepared micellar prodigiosin, by adding surfactants during the fermentation process and then complexed it with metal ions, in order to tackle the lack of affinity of cellulosic substrates [31]. Wu et al. also prepared prodigiosin micelles to dye a chemically modified lyocell fabric to improve color retention [34]. It is worth mentioning that all prodigiosin-functionalized fabrics produced by these authors had enhanced antibacterial activity against a plethora of pathogens. The low aqueous solubility of prodigiosin in textile dyeing is considered a major limitation. Consequently, various approaches have been developed based on solvent-free dyeing techniques and the optimization of processing parameters [36].

In this study, prodigiosin production was optimized in *P. putida* strain PIG02, comparing rich and minimal medium, and extracted from cells into solid crude extracts for solvent-free textile dyeing and printing processes. In dyeing assays, the textile substrates selected were CO and PES fabrics since these fibers are the most consumed in the textile industry. The effect of prodigiosin’s protonation state on the fabric’s functionalization was tested by preparing prodigiosin suspensions in acid, alkaline, and neutral buffer solutions. Moreover, six surfactants (two anionic, two cationic, and two non-ionic) were incorporated to test and improve prodigiosin’s solubility in aqueous environments, in order to avoid using ethanol or other organic solvents. Chitosan (Ch) and tannic acid (TA) were tested as biomordants for the CO dyeing processes, evaluating their ability to replace metallic mordants. Prodigiosin was also unprecedentedly tested in printing processes, where two biopolymers were used, cellulose acetate and sodium alginate, to evaluate their ability to retain color. A conventional printing thickener (Gilaba FF) was also used for comparative purposes. Dyed and printed fabrics were further characterized by determination of CIELab* color coordinates, Fourier transform infrared spectroscopy (FTIR), Scanning Electron Microscopy (SEM), Energy-Dispersive Spectroscopy (SEM-EDS), swelling properties, as well as light, rubbing, and washing fastness. Furthermore, considering the reported properties of prodigiosin, halochromic properties and antimicrobial potential of prodigiosin-dyed and prodigiosin-printed fabrics were tested.

## 2. Materials and Methods

### 2.1. Materials and Chemicals

The culture media Lysogenic Broth Agar (maintenance agar medium) and Terrific Broth (rich medium), used in prodigiosin production experiments (as lyophilized media), non-baffled culture flasks, 0.22 μm nylon syringe filters, chloroform, acetic acid glacial, cetyltrimethylammonium bromide cationic surfactant (CTAB), and agar were obtained from VWR Chemicals (Carnaxide, Oeiras, Portugal). The components of the mineral salts medium (minimal nutritional conditions; 8.9 mM potassium phosphate dibasic, 6.2 mM sodium dihydrogen phosphate, 34.2 μM ethylenediaminetetraacetic acid, 7 μM zinc sulfate, 6.8 μM calcium chloride, 18 μM iron (II) sulfate, 0.8 μM sodium molybdate, 0.7 μM copper sulfate, 1.7 μM cobalt chloride, 1.9 μM manganese chloride, 15.1 mM ammonium sulfate and 0.5 mM magnesium chloride), ethanol (absolute), isopropyl β-D-1-thiogalactopyranoside (IPTG), benzalkonium chloride (alkylbenzyldimethylammonium, BC), sodium sulfate, and sodium chloride were purchased from Merck (Lisboa, Portugal). Tryptic Soy Broth (TSB) and Tryptic Soy Agar (TSA), used in antimicrobial assays, were purchased from Liofilchem (Teramo, Italy). Glycerol, lactic acid, and sodium phosphate dibasic were purchased from Fisher Scientific (Hampton, VA, USA). Commercial prodigiosin standard was acquired from Cayman Chemicals (≥95% purity). Triton X-100, sodium dodecyl sulfate (SDS) (non-ionic and anionic surfactant, respectively), Ch high molecular weight, sodium alginate, cellulose acetate (MW 30,000), tris-hydrochloride, disodium hydrogen phosphate, and potassium phosphate monobasic were purchased from Sigma-Aldrich Química S.L. (Lisboa, Portugal). Tween 80 non-ionic surfactant, hydrochloric acid (HCl), sodium phosphate dibasic, and potassium chloride were acquired from Panreac (Barcelona, Spain). TA was obtained from Alfa Aesar (Ward Hill, MA, USA). Gilaba FF thickener was purchased from Gilaba—Máquinas e Produtos Químicos (Porto, Portugal). Sodium acetate anhydrous was acquired from Carlo Erba Reagents GmbH (Barcelona, Spain), Diadavin UN from Tanatex Chemicals (Santo Tirso, Portugal), and sodium dioctyl sulphosuccinate anionic surfactant (Lutensit A-BO) from BASF (Porto, Portugal).

Commercial pre-washed CO (Lameirinho—Indústria Têxtil S.A., Guimarães, Portugal), PES and PA (Lemar-Leandro Magalhães de Araújo (Filhos) Lda, Guimarães, Portugal), SK (Acatel-Acabamentos Têxteis S.A., Barcelos, Portugal), and WO (Valérius-Têxteis S.A., Barcelos, Portugal) fabrics were used for dyeing/printing tests (Table 1). Multifiber adjacent Deutsche Welle (DW) fabric and ECE phosphate reference detergent were purchased from SDC Enterprises Ltd. (Thongsbridge, West Yorkshire, UK).

### 2.2. Bacterial Strains and Culture Media

*Pseudomonas putida* strain PIG02 was kindly provided by Dr. Brian Pfleger for the production of prodigiosin [25]. The strain was maintained in LBA plates up to 3 passages for each production experiment. Prodigiosin production was carried out in either rich Terrific Broth supplemented with 271 mM glycerol (2.5% (*v*/*v*)), herein designated as TB-G [37], or mineral salts medium [38] supplemented with 271 mM glycerol and 48 mM lactic acid, herein MM-G. *Escherichia coli* ATCC 25922, *Pseudomonas aeruginosa* ATCC 27853, and *Staphylococcus aureus* ATCC 6538 were acquired from the American Type Culture Collection and used in the antimicrobial assays.

### 2.3. Testing Conditions for Prodigiosin Production

Prodigiosin production was tested in both TB-G (rich medium) and MM-G (minimal medium), at 30 °C and 200 rpm, up to 72 h of biosynthesis. Pre-cultures were grown overnight from single colonies of LB agar plates, in non-baffled 100 mL flasks with 20 mL of TB-G or MM-G, to inoculate production cultures of 100 mL in non-baffled 500 mL flasks, with an initial optical density at 600 nm (OD600 nm) of 0.1. When cultures reached OD600 nm values of 1.0, prodigiosin production was induced by adding IPTG to a final concentration of 1.0 mM. Levels of intracellular prodigiosin were quantified at 4, 24, 30, 48, 54, and 72 h after inducing the biosynthesis, by collecting 1.0 mL of culture in duplicate, from 4 independent experiments. Cells were centrifuged at 8000× *g* and 4 °C, for 15 min, and the biomass was washed twice with 0.9 wt% NaCl solution. Prodigiosin accumulated in the cells was extracted with 40 mM HCl in absolute ethanol, after resuspending and vortexing the biomass for 10 min at 1200 rpm in a MultiVortex MSV-3500 (Paralab, Porto, Portugal), and the red supernatant collected and cleared by centrifugation at 14,000× *g* and 4 °C, for 20 min, in a Heraeus Multifuge 1 L-R (Thermo Scientific, Waltham, MA, USA). The extraction was repeated until the supernatant was colorless. The total amount of prodigiosin was estimated as the sum of recovered pigment, expressed as mass per volume of culture. The prodigiosin-rich supernatant was filtered through a 0.22 μm nylon membrane, and prodigiosin was quantified using a Shimadzu UV-2700 spectrophotometer (Shimadzu Europa GmbH, Duisburg, Germany). The peak of maximum absorbance at 534 nm was identified, and the UV–Vis spectra of the samples were compared with those of commercial prodigiosin standards at different concentrations, all prepared in the same solvent. A calibration curve was generated with 1.0, 2.5, 5.0, 10, 20, and 50 µg/mL of prodigiosin.

### 2.4. Production of Crude Prodigiosin Biopigment

The crude prodigiosin extracts to be used in textile assays were produced from *P. putida* PIG02 cells grown in MM-G, as aforementioned. After 72 h of prodigiosin production, 40 mL aliquots of bacterial cells were centrifuged in 50 mL centrifuge tubes at 8000× *g* and 4 °C, for 15 min, and washed twice with 0.90 wt% NaCl. Prodigiosin was extracted from cells, filtered, and quantified as described in the previous section, repeating the extraction until the supernatant was colorless.

The solvent of the crude extracts was removed under reduced pressure by rotatory evaporation in a Büchi R-100 Rotavapor (Uster, Switzerland), at 100 mbar, with a water bath at 40 °C. The concentrated solid residue was washed twice with (i) 5 M NaCl, (ii) 250 mM phosphate buffer pH = 7 containing 5 M NaCl, or (iii) 250 mM Tris-HCl pH = 10 containing 5 M NaCl, to remove water-soluble impurities in acid, neutral, and alkaline conditions, respectively. Afterwards, the red solid of each washing condition was solubilized in chloroform, and the amount of solid prodigiosin extract necessary for the textile assays was dried by a nitrogen stream to remove the solvent. Prodigiosin extracts were analyzed using UV–Vis spectroscopy and high-performance liquid chromatography with diode array detectors coupled to mass spectrometry (HPLC-DAD-MS), using commercial prodigiosin as standard.

### 2.5. High-Performance Liquid Chromatography of Prodigiosin Crude Biopigment

HPLC-DAD-MS was used for the qualitative analysis of the composition of the crude extract, in comparison to commercial prodigiosin. Aliquots of extracted prodigiosin were dried under a nitrogen stream and resolubilized in ethanol with 1.0% (*v*/*v*) formic acid, to a final concentration of 0.1 mg/mL for the analysis. Samples were filtered through 0.22 µm polytetrafluoroethylene syringe filters, and 10 µL were injected into a Waters Alliance 2695 liquid chromatography equipment with a Waters SunFireTM C18 column (2.1 mm × 100 mm, 5 µm) and a triple quadrupole MicroMass Quattromicro API mass spectrometer (Waters, Ireland). Elution was performed in isocratic mode with 1.0% mobile phase A (0.10% (*v*/*v*) formic acid in water) and 99% mobile phase B (acetonitrile), at a flow rate of 0.3 mL/min and a column temperature of 35 °C. The extract composition was analyzed in Full Scan mode, spectrophotometrically in the wavelength range of 210 nm to 790 nm. The ionization was carried out by electrospray in both positive and negative ion modes, at 30 V. MassLynx software v. 4.1 was used for data acquisition and processing.

### 2.6. UV–Vis Spectroscopy of Prodigiosin Solution

The neutral prodigiosin extract was solubilized in absolute ethanol for UV–Vis spectroscopy analysis (Shimadzu UV-2600, Shimadzu Corporation, Kyoto, Japan). Absorbance curves were evaluated at different pH values: acidic (pH = 3), neutral (pH = 7), and basic (pH = 13).

### 2.7. Textile Dyeing and Printing with Prodigiosin Biopigment

#### 2.7.1. Functionalization of Textiles with Prodigiosin Extract Through Dyeing Process

The different fabrics (CO, PES, SK, WO, and multifiber adjacent fabric) were dyed using an Ibelus C-720 (Pregitzer & Ca., Lda, Guimarães, Portugal) equipped with an infrared heating system, with a rotation of 50 rpm and 50 cycles. The dyeing process was optimized for each type of fiber:

(i) Multifiber adjacent fabric, SK, and CO: heating at a rate of 2.0 °C/min to 80 °C, maintained at this temperature for 60 min, followed by cooling to 60 °C at a rate of 3.5 °C/min;
(ii) PES: heating in stages to 60 °C (3 °C/min, maintained for 10 min), followed by 90 °C (2 °C/min, maintained for 30 min) to 130 °C (1.5 °C/min, maintained for 45 min), with further cooling in two stages: from 130 to 80 °C (2 °C/min) and from 80 to 30 °C (2.5 °C/min);
(iii) PA and WO: heating at 100 °C (2 °C/min, maintained for 60 min), followed by cooling to 60 °C (2 °C/min).

All the dyeing processes used a concentration of 1.25 g/L of prodigiosin biopigment, with the different washing processes (acidic, neutral, and alkaline batch), and a bath ratio of 1:20. Then, samples were washed with distilled water, followed by a washing with Diadavin UN detergent (1 g/L) at boiling temperature, for 20 min, to remove any unbonded pigment and residual surfactant, and dried in an oven at 40 °C.

#### 2.7.2. CO and PES Printing with Prodigiosin Biopigment and Biopolymers

Based on the results obtained during the application of the biopigment by exhaustion (semi-continuous dyeing), CO and PES fabrics, as well as neutral and alkaline batches, were selected for application via conventional printing. The printing process employed a Zimmer Mini MDF R541 table (Promatex, Porto, Portugal), equipped with a 107 threads per inch screen mesh and 6 mm rod-squeegee, operated at a speed of 15.5 m/s and magnetic pressure of 3 (on a scale of 1 to 6, where 1 is the lowest pressure and 6 the highest), performing two passes (backward and forward) of the printing paste. Color transfer from an intermediate to the textile fabric was performed using an open screen mesh. Prodigiosin at 1.25 g/L (concentration used in exhaustion dyeing) was first dissolved in 1 mL of 96% ethanol and subsequently added to 10 g of Gilaba FF thickener/sodium alginate (2 wt%)/cellulose acetate (15 wt%) printing paste. Thus, a comparison between the commercial paste and natural polymers was carried out. All printed materials with the commercial thickener and the cellulose acetate formulation were dried at 100 °C by performing 2 passages at 3 m/min on the Model E 20 drying mat (S. Roque, Portugal), followed by thermosetting on the Stenter Werner Mathis AG roller (São Paulo, Brazil) at 150 °C for 3 min. Samples prepared with sodium alginate were immersed in a 2 wt% CaCl_2_ solution (crosslinker) post-printing, followed by rinsing in distilled water and drying at 60 °C for 2 h.

### 2.8. Preparation of Buffers for Dyeing Multifiber and Fabrics

To identify the fibers with the highest affinity for the prodigiosin extract, a solubility study of the acidic pigment was conducted, followed by dyeing of multifiber DW and SK fabrics as described in Section 2.7.1. The solubility assay was performed using different ethanol systems, namely, ethanol:distilled water (dH_2_O) 1:1, and three different buffers: ethanol:acetate buffer (pH = 4), ethanol:phosphate buffer (pH = 7), and ethanol:tris-hydrochloride buffer (pH = 9), all 1:1 ratio. Following optimization, PES, PA, and WO fabrics were dyed with ethanol and ethanol-based systems, with the exception of the ethanol:acetate buffer system, which was not applied to PA and WO fabrics.

### 2.9. Evaluating Surfactant Effects on Dye Solubility and CO and PES Dyeing Process with Acidic Prodogiosin

Six different surfactants at their critical micelle concentration (CMC) (room temperature, dH_2_O) were selected to replace ethanol in the dyeing of multifiber DW fabric, without compromising pigment solubility. The surfactants evaluated included anionic (Lutensit and SDS), non-ionic (Triton X-100 and Tween 80), and cationic (CTAB and BC) types. The dyeing solutions were prepared with the following concentrations: 0.9 mM Triton X-100 (pH = 7.06) [39], 0.02 mM Tween 80 (pH = 5.26) [40], 2.66 mM Lutensit (pH = 4.75) [41], 8.2 mM SDS (pH = 4.30) [39], 0.9 mM CTAB (pH = 3.82) [39], and 5.2 mM BC (pH = 4.16 for n=18) [42,43]. These solutions were emulsified using a T25 easy clean Ultra-Turrax disperser (IKA-Werke GmbH & Co. KG, Staufen, Germany) at 10,000 rpm for 2 min, to promote the formation of a uniform suspension of the biopigment, given the poor water solubility.

Moreover, CO and PES fabrics were dyed only with the non-ionic surfactants Triton X-100 (0.9 mM) and Tween 80 (0.02 mM), and the cationic surfactants CTAB (0.9 mM) and BC (5.2 mM), as detailed in Section 2.7.1. The pH values of the dyeing solutions ranged from 3.5 to 3.9.

### 2.10. Optimization of Dyeing with Non-Ionic Surfactants and Acidic Prodogiosin

After identifying the most suitable surfactants for dyeing CO and PES fabrics, the dyeing process was optimized by adjusting the surfactant concentrations relative to their CMC. For each non-ionic surfactant applied to CO fabrics, one concentration below the CMC was tested: 0.36 mM for Triton X-100 and 0.008 mM for Tween 80. Additionally, two concentrations above the CMC were evaluated: 1.44 and 1.98 mM for Triton X-100, and 0.32 and 0.44 mM for Tween 80. For PES fabrics, only the concentrations optimized for CO fabrics were used, namely, 0.36 and 1.44 mM of Triton X-100, and 0.008 and 0.32 mM of Tween 80. The aqueous solutions were emulsified using a T25 easy clean Ultra-Turrax disperser (10,000 rpm) for 1 to 2 min.

### 2.11. Evaluation of Dyeing Performance in CO and PES Fabrics Under Alkaline and Neutral Prodigiosin

Following the determination of the optimal application conditions for the acidic batch applied to CO and PES fabrics, these optimized parameters were subsequently evaluated under alkaline and neutral batches for CO, PES, and multifiber DW fabrics. For comparative purposes, the multifiber DW fabric was subjected to the dyeing conditions previously applied to the individual CO and PES fabrics (Section 2.7.1). The aqueous solutions were emulsified using a T25 easy clean Ultra-Turrax disperser, at 10,000 rpm, for 1 to 2 min.

### 2.12. Mordanting Process of CO Fabric Under Alkaline and Neutral Prodigiosin

CO substrates dyed with prodigiosin from the neutral batch, under the optimal concentration of Tween 80, were subjected to biological mordanting, either prior to or following the dyeing process. In the pre-mordanting method, the CO fabric was treated in a mordanting bath composed of a 2% over fabric weight TA solution, using a liquor ratio of 1:20, for 1 h at 90 °C. Subsequently, the CO sample was dyed through the method described in Section 2.7.1. For the post-mordanting method, the previously dyed sample was immersed in a solution containing 2 wt% Ch dissolved in acetic acid at 2% (*v*/*v*) (pH = 3.8). After dipping, the specimen was passed through a two-roller foulard (Roaches, UK) with a liquor pick-up of 80%, followed by drying at 90 °C for 2.5 min and thermosetting at 110 °C for 3 min.

### 2.13. Characterization of Functionalized Fabrics

#### 2.13.1. CIELab* Coordinates and Color Strength (K/S)

K/S values and color coordinates were obtained using a UV-2600 spectrophotometer (Shimadzu Europa GmbH, Duisburg, Germany). The reflectance spectra of the fabrics functionalized with prodigiosin were analyzed in 400–700 nm range, at 1 nm intervals. Color coordinates were obtained using the D65 illuminant and a 10-degree observation angle, according to the Commission Internationale de l’Eclairage (CIELab), where L* corresponds to lightness, a* is relative to red-green (+red, −green), and b* indicates yellow-blue (+yellow, −blue).

The color difference (ΔE) was calculated using Equation (1), where ΔL* is the lightness difference, and Δa* and Δb* are the differences between red-green and yellow-blue, respectively. It is noteworthy that ΔE values exceeding 1 are generally perceptible to the human eye [44]. The K/S of the functionalized samples was determined based on the Kubelka–Munk Equation (2), where K is the absorption coefficient, S is the scattering coefficient, and R is the decimal fraction of the reflectance. The values correspond to the average of three consecutive measurements taken at different positions on the fabric surface.

(1) $$\Delta E=\sqrt{{{(∆L}^{*})}^{2}+{{(∆a}^{*})}^{2}+{{(∆b}^{*})}^{2}}$$

(2) $$K/S={\left(1-R\right)}^{2}/2R$$

#### 2.13.2. Washing Fastness

The washing fastness of the dyed and printed samples was evaluated based on an adaptation of the ISO 105-C06:2010 standard (Tests for color fastness—Color fastness to domestic and commercial laundering). Briefly, samples (4 × 10 cm) were sewn to the multifiber adjacent fabric and immersed in 150 mL of washing bath containing 4 g/L of ECE detergent. The washing procedure was evaluated at 70 °C for 30 min using an Ibelus C-720 machine. Subsequently, samples were rinsed with distilled water and dried in an oven at 40 °C. Washing fastness was assessed through UV–Vis spectroscopy by calculating the ΔE Equation (1) and K/S Equation (2). In addition, staining on both the fabric and adjacent multifiber fabric was evaluated using the gray scale (ranging from 1, indicating poor fastness, to 5, indicating excellent fastness). Reflectance spectra were obtained by UV–Vis spectroscopy in the wavelength range of 400–700 nm, with a resolution of 1 nm. Reported values correspond to the average of three consecutive measurements performed at different positions. The evaluation of color change and staining was conducted in accordance with ISO 105-A02:1993 and ISO 105-A03:1993 standards, respectively.

#### 2.13.3. Light Fastness

To evaluate UV light fastness, the functionalized samples were exposed to UV radiation for 2 and 4 h at 50 °C, without condensation. The assay was performed using QUV equipment (Q-Lab, Westlake, OH, USA). The evaluation of change in color and staining was performed according to ISO 105-A02 and ISO 105-A03, respectively. Subsequently, ΔE and K/S were determined using Equations (1) and (2), respectively. The corresponding reflectance spectra were obtained by UV–Vis spectroscopy, according to Section 2.13.1.

#### 2.13.4. Rubbing Fastness

Fastness to dry and wet rubbing was evaluated according to the standard ISO 105-X12:2016. Briefly, functionalized samples (7 × 14 cm) were placed in the crockmeter (Roaches, West Yorkshire, UK), and a CO rubbing cloth (5 × 5 cm), either dry or wet, according to ISO 105-F09:2009, was used as the test fabric. The CO rubbing cloth performed 10 rubbing cycles in alternating directions on the surface of the functionalized samples, under a pressure of 9 N and at a speed of 1 cycle/s. The evaluation of change in color and staining was performed according to ISO 105-A02 and ISO 105-A03, respectively.

#### 2.13.5. Halochromic Properties of the Functionalized Fabrics with Neutral Prodigiosin

The halochromic properties and color reversibility of the optimized dyed and printed samples were systematically evaluated using acid (HCl 10% *v*/*v*) or alkaline (NaOH 10 wt%) solutions. Cycles of pH variation were performed (1 and 10; a cycle is defined as dipping the fabric in NaOH and HCl), during which the necessary time for color reversibility was recorded. Furthermore, visual documentation was captured at each stage to enable a comprehensive analysis of the color changes and the efficiency of the reversibility process.

#### 2.13.6. Swelling Determination

The optimized dyed and printed samples were subjected to a swelling assay to evaluate their liquid absorption capacity, a parameter of relevance for wound dressing. Samples with 1 cm^2^ were immersed in 5 mL of phosphate-buffered saline (PBS, pH 7.4) at 37 °C, under thermophysiological conditions. Agitation was maintained at 50 min^−1^ using a VLSB18 shaking bath (VWR Chemicals, Carnaxide, Portugal) [45,46]. The swelling percentage was quantified at various times (0.25, 0.50, 0.75, 1, 2, 4, 6, 24, 48, and 72 h) according to Equation (3). Before weighing, excess surface solution was carefully removed using Kimwipes™ Delicate Task Wipers (Irving, TX, USA). All measurements were performed in triplicate.

(3) Swelling (%) = [(W_t_ − W_i_)/W_i_] × 100

where W_i_ is the initial weight, and W_t_ is the weight at each time point.

#### 2.13.7. Attenuated Total Reflectance—Fourier Transform Infrared Spectroscopy (ATR-FTIR)

Fourier transform infrared (FTIR) spectroscopy, equipped with a horizontal Attenuated Total Reflectance (ATR) accessory (Norleq, Porto, Portugal), was employed for a chemical characterization of the optimized dyed and printed samples. Spectra data were acquired with 64 scans at a resolution of 4 cm^−1^, within the spectral range of 4000 to 600 cm^−1^.

#### 2.13.8. Scanning Electron Microscopy (SEM) and Energy-Dispersive Spectroscopy (EDS)

Morphological characterization of the optimized dyed and printed samples was performed using scanning electron microscopy (SEM) with a FlexSEM 1000 microscope (Hitachi, Tokyo, Japan) operating at an accelerating voltage of 10 kV. Prior to image acquisition, the functionalized samples were mounted on conductive carbon adhesive tape and sputter-coated with a gold layer of 7.5 nm for 90 s, using a Quorum Mini QS coater (London, UK). Elementary identification and composition (in weight, %) was performed through EDS analysis using a Quantax 75 detector (Hitachi, Tokyo, Japan), equipped with ESPRIT compact software (version 2.3.0.1017), at 15 kV.

#### 2.13.9. Antimicrobial Activity

Samples of CO and PES dyed with the neutral and alkaline batches in the presence of the surfactant Tween 80, at the optimized concentration of 0.44 mM, as well as their respective controls, were evaluated. In addition, CO and PES samples printed either with the commercial paste Gilaba FF or the cellulose acetate solution containing the neutral biopigment batch, and their respective controls were assessed. Samples measuring 2 × 2 cm were subjected to an antimicrobial assay according to AATCC TM-100 standard. Briefly, pre-inocula of *S. aureus*, *E. coli*, and *P. aeruginosa* were prepared in TSB and incubated overnight at 37 °C and 120 rpm. Subsequently, the fabrics were incubated with 50 µL of a sterilized phosphate-buffered saline (PBS) solution containing 1 × 10^7^ colony-forming units (CFU)/mL of each bacteria, at room temperature (≈21 °C), for 1 h. Samples were then transferred to 5 mL of a PBS solution and subjected to vortex agitation for 1 min. This solution was then exposed to 1:10 serial dilutions, each of which was plated on a solid medium (TSA). These plates were incubated for 16 h, at 37 °C, with 90% humidity. The CFUs were counted, and the logarithmic reduction (log) was calculated according to Equation (4). The assay was carried out in quadruplicate for each sample and microorganism. The antimicrobial properties of the functionalized fabrics were classified qualitatively according to the criteria established by Vieira et al. [47].

(4) Log reduction = Log (control) − Log (exposed)

where control is the inoculum concentration of the respective microorganism, and exposed is the concentration of the microorganism in fabrics after 1 h of incubation.

#### 2.13.10. Statistical Analysis

The statistical analysis of antimicrobial activity was performed using GraphPad Prism software v. 9.5.1 (San Diego, CA, USA) for MacBook. The normality of the data distribution was evaluated using the Shapiro–Wilk test. For data exhibiting normal distribution, statistical comparisons were performed using two-way analysis of variance (ANOVA), followed by Tukey’s multiple comparison test for intra-fabric analyses, and Šídák’s multiple comparison test for inter-substrate comparisons. For data that did not follow a normal distribution, the non-parametric Kruskal–Wallis test was applied, followed by Dunn’s multiple comparisons test (post hoc test). Statistical comparisons were reported exclusively for group pairs deemed methodologically appropriate. All *p*-values below 0.05 were considered statistically significant. Results are reported as mean ± standard deviation.

#### 2.13.11. Photographs

Digital photographs of prodigiosin-functionalized fabrics were taken with a smartphone Samsung S24 Ultra, model number SM-S928B/DS, whereas images of the pristine substrates were obtained using a magnifying glass (Leica EZ4D, Carnaxide, Oeiras, Portugal). Images of functionalized fabrics were acquired in a light chamber, under a D65 light source. No adjustments of pixels, color, brightness, or contrast were applied to the images. The photographs produced were parts of larger images that were selected without obscuring, eliminating, or misrepresenting any information present in the originals.

## 3. Results and Discussion

### 3.1. Production, Extraction, and Characterization of Prodigiosin Crude Extracts

The heterologous production of prodigiosin was carried out in *P. putida* PIG02 [25]. The genes coding for the biosynthetic pathway are integrated into the chromosome of the strain, as part of an expression system under the control of the IPTG-inducible Ptrc promoter [25]. The production of prodigiosin was tested both in rich and minimal media to assess the best nutritional conditions for the biosynthesis. Both media were supplemented with glycerol as the main carbon source, because it has been the most commonly used compound supporting prodigiosin biosynthesis [25,26].

**Figure 2:**
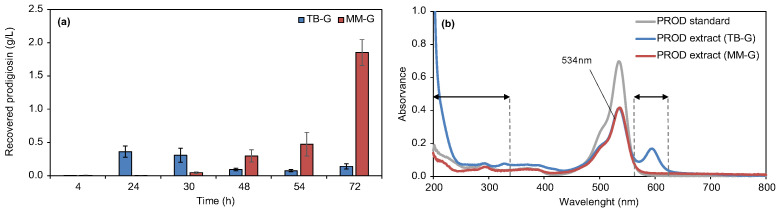
Production of prodigiosin by *P. putida* PIG02, over 72 h of biosynthesis in rich and minimal media. (**a**) Amount of prodigiosin recovered from cells grown in rich Terrific Broth medium supplemented with 271 mM glycerol (TB-G; blue), or in minimal medium supplemented with 271 mM glycerol and 48 mM lactic acid (MM-G; red), at 30 °C and 200 rpm. Prodigiosin was extracted in 40 mM HCl in ethanol and quantified by the maximum absorbance at 534 nm. The amount of prodigiosin extracted is expressed as concentration per volume of culture. (**b**) Representative UV–Vis spectra of the crude extracts obtained from TB-G (blue) and MM-G (red), and of the 10 µg/mL prodigiosin standard (gray). Black arrows and dotted gray lines highlight ranges of wavelength in which the spectra of extracts and standard differ (200–350 nm; 563–610 nm).

Prodigiosin production was monitored at 4, 24, 30, 48, 54, and 72 h to determine the biosynthesis time point resulting in a higher yield of recovered biopigment. A maximum of 1.85 ± 0.19 g/L (5.70 ± 0.60 mM) was obtained in MM-G at 72 h after inducing biosynthesis with 1 mM IPTG, compared to the maximum of 0.36 ± 0.09 g/L (1.10 ± 0.30 mM) recovered from TB-G at 24 h (Figure 2a).

The absorbance spectra of the prodigiosin extracts from MM-G showed great similarity with the spectrum of the prodigiosin standard (Figure 2b), with the maximum absorbance peak at 534 nm. This suggested a low level of contaminants in the extract. Prodigiosin extracts from TB-G presented additional unique absorbance peaks in the spectra, at 331 and 598 nm, and higher absorbance between 200 and 242 nm. Although we did not aim at identifying the sources of differences in the absorbance spectra, these unique peaks, not existing in the standard, clearly suggested the presence of additional components in the extract derived from TB-G productions. Prodigiosin biosynthesis in MM-G for 72 h, at 30 °C and 200 rpm, was thus selected as the most promising culture conditions for the production of crude extract with higher yields, for the following textile dyeing assays.

Terrific Broth supplemented with glycerol has been used previously to produce prodigiosin by *P. putida* strains. A maximum of 0.15 g/L (0.50 mM) of prodigiosin was obtained with *P. putida* pig21 constitutively expressing the biosynthetic pig gene cluster and growing in TB supplemented with 55 mM glycerol [26]. Moreover, Cook and co-workers compared prodigiosin production in both rich and minimal nutritional conditions in strain PIG02 [25]. These authors demonstrated that production in Riesenberg–Korz minimal medium supplemented with 271 mM glycerol resulted in a higher prodigiosin yield of 1.10 g/L (3.4 mM), compared to the 0.50 g/L (1.5 mM) obtained in cultures with the rich medium TB supplemented with the same amount of glycerol. In this study, a 68% higher yield of prodigiosin was obtained at the laboratory scale with strain PIG02 under minimal nutritional conditions (1.85 vs. 1.10 g/L).

**Figure 3:**
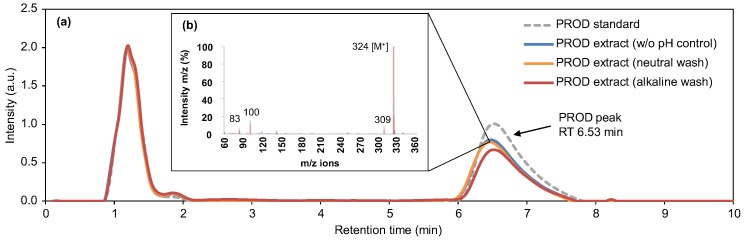
HPLC-DAD-MS analysis of prodigiosin (PROD) crude extracts produced from *P. putida* PIG02 in minimal medium supplemented with 271 mM glycerol and 48 mM lactic acid (MM-G). (**a**) Overlay of representative chromatograms of crude extracts washed with 5 M NaCl (blue line), 250 mM phosphate buffer pH = 7 with 5 M NaCl (orange line), 250 mM Tris-HCl pH = 10 with 5 M NaCl (red line), and commercial prodigiosin (dashed gray line), detected spectrophotometrically by a diode array detector at 210–790 nm. (**b**) Overlay of representative mass spectra of the prodigiosin peaks at RT 6.53 min. Crude extract from acid wash is shown in purple and commercial prodigiosin in gray.

To obtain crude prodigiosin extract in laboratory production at a larger scale, prodigiosin extracted with 40 mM HCl in ethanol was concentrated by rotary evaporation at 100 mbar. The solid residue was washed with 3 different solutions: 5 M NaCl, 250 mM phosphate buffer pH = 7 containing 5 M NaCl, or 250 mM Tris-HCl pH = 10 containing 5 M NaCl, to remove water-soluble impurities and obtain a final solid prodigiosin batch in acid (pH not controlled), neutral, and alkaline conditions, respectively. Crude extracts were qualitatively analyzed using HPLC-DAD-MS before final drying under a nitrogen stream (Figure 3).

The chromatogram of the crude prodigiosin extracts showed similar profiles among wash conditions and when compared to the commercial prodigiosin (Figure 3a). Under the detection methodology used, prodigiosin was the major component, detected at the retention time of 6.53 min (excluding the peaks of the solvents ethanol and formic acid at 1.18 and 1.86 min). Prodigiosin identification was validated by comparison with the chromatogram and MS spectrum of the standard, with the detection of the signature parental ion of *m/z* 324 (M = 323 g/mol), and key ions of *m/z* 309, 100, and 83 (Figure 3b). Thus, the prodigiosin produced in this work can be compared to prodigiosin produced by other bacterial strains. Other cellular components with strong UV absorbance (e.g., proteins) were not detected by analyzing the absorbance spectra of the samples in the wavelength range of 210 to 290 nm.

**Figure 4:**
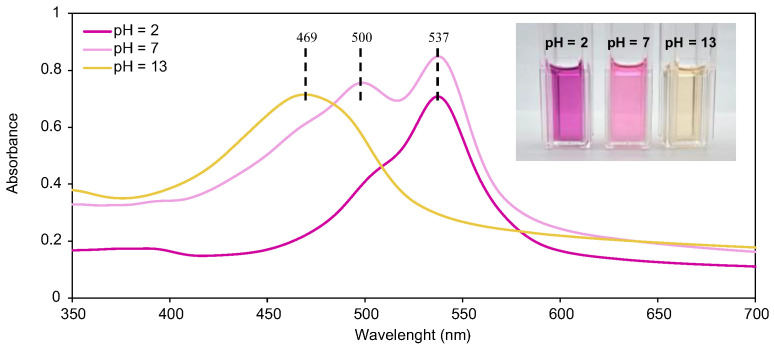
The acid–base equilibrium of prodigiosin solutions at different pH values, characterized by UV–Vis spectroscopy.

Following HPLC purification, the UV–Vis spectra of prodigiosin at different pHs were obtained (Figure 4). The absorbance profile of prodigiosin was characterized, and pH-dependent structural and chromatic variations were identified, which are considered essential for the understanding of its behavior and potential application in pH-responsive wound dressings. For prodigiosin produced by *P. putida* PIG02, maximum absorbance was observed at 537 nm for acidic conditions, 500 and 537 nm for neutral conditions, and 469 nm for alkaline conditions. The maximum wavelength at neutral and acidic pH is described in the literature between 533 and 540 nm, while at basic pH, it ranges from 465 to 470 nm for various producing strains, including *Serratia marcescens*, *Serratia nematodiphila*, and *Pseudoalteromonas rubra* [32,35,48,49,50].

At a laboratory scale, the production cost of prodigiosin is estimated to have a value of 33.50 €/g. Nevertheless, it should be taken into consideration that this value may change considerably upon changing the production scale, or reagents and consumables quality grade.

### 3.2. Application and Optimization of the Biopigment and Color Fastness Assays

#### 3.2.1. Evaluation of Surfactants’ Effect on Prodigiosin Solubility and CO and PES Dyeing Process Under Acidic Biopigment

CO and PES fibers, being the most prevalent in the textile industry, were selected for investigation involving the incorporation of prodigiosin [51]. A comparative study was conducted using mechanical dispersion to apply non-ionic and cationic surfactants as emulsifiers. The formation of an emulsion enhanced the stability of biopigment and dyeability by promoting particle fragmentation, thereby reducing spot and agglomerate formation on fabric surfaces. These improvements led to enhanced results with ethanol-based solvent systems and cost reductions (Supporting Information, S.I., Tables S1–S4; Figure S1) [17,52].

**Figure 5:**
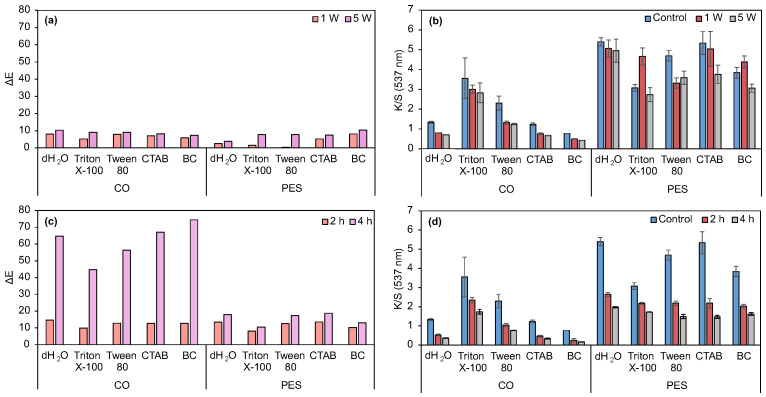
ΔE for washing (**a**) and UV light fastness (**c**) and K/S for washing (**b**) and UV light fastness (**d**) of dyed CO and PES fabrics with acidic prodigiosin and non-ionic and anionic surfactants.

Dyeing processes revealed that non-ionic surfactants produced superior color performance on both fabrics (Table S5). Prodigiosin demonstrated greater affinity for PES, as indicated by higher K/S values in control samples compared to CO, irrespective of surfactant presence (Figure 5c,d) [27,30]. Washing fastness tests showed PES to be most promising except when cationic surfactants were employed. Non-ionic surfactants improved ΔE after one wash, especially for CO, although this effect diminished after five cycles. No considerable differences in light fastness were observed between substrates or modifications (Figure 5b,d). Notably, an additive effect was observed for PES and prodigiosin after 4 h of light exposure, with lower ΔE values and better performance upon non-ionic surfactant application. While surfactants are commonly applied during dye exhaustion, literature also reports their use in fermentation to enhance micelle formation and pigment yield [53,54]. Ren et al. demonstrated that non-ionic surfactants (Tween 80 and Triton X-100) provided superior micelle production yields and extraction efficiency compared to anionic surfactants (SDS and dodecyl benzene sulfonate) [16]. Therefore, an evaluation of the optimal concentration of non-ionic surfactant to be used was conducted to evaluate washing and light fastness.

**Figure 6:**
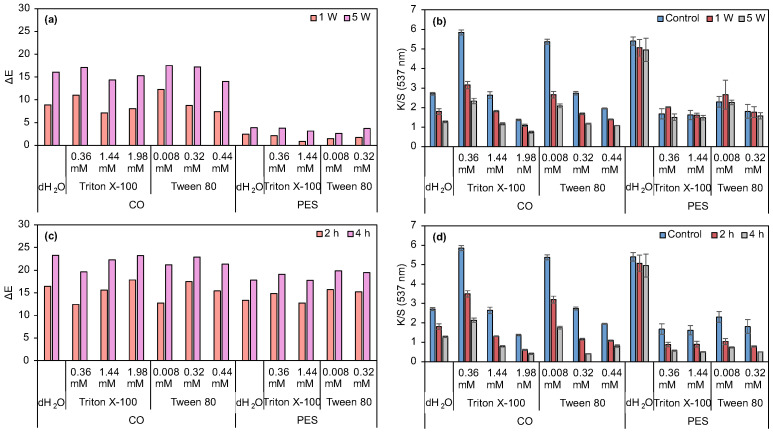
ΔE for washing (**a**) and UV light fastness (**c**) and K/S for washing (**b**) and UV light fastness (**d**) of dyed CO and PES fabrics dyed with acidic prodigiosin and different concentrations of Triton X-100 and Tween 80.

Functionalized PES fabric demonstrates superior performance in the water fastness assay, as well as excellent dry and wet rubbing fastness (Table S6). These results highlight the enhanced durability and stability of PES fabric under these conditions. Optimization revealed that non-ionic surfactants markedly improved prodigiosin dispersion, enhancing color intensity and uniformity. Concentrations both below and above the CMC were tested: 0.36, 1.44, and 1.98 mM for Triton X-100, and 0.008, 0.32, and 0.44 mM for Tween 80, to maximize pigment stability and dispersion. Mechanical dispersion via Ultra-Turrax yielded higher K/S values, though surfactant addition reduced K/S with minimal ΔE variation after 4 h of light exposure (Figure 6c,d). PES showed higher K/S and lower ΔE in washing tests, whereas CO fabrics exhibited increased K/S at surfactant concentrations below CMC (0.008 mM Tween 80 and 0.36 mM Triton X-100), resulting in darker shades (Table S7). Reduced differences in K/S and ΔE between controls and treated samples suggest stronger dye-fiber affinity, with higher surfactant concentrations enhancing dye stability despite initially lowering K/S values, likely due to improved pigment dispersion and stability compared to sub-CMC concentrations [55]. Gray scale evaluation indicated excellent washing fastness for PES, with a majority of color change ratings of 4 or 5 and excellent staining (4/5 and 5, Table S8). Regarding CO fabric, no staining was detected after the first wash cycle, except for the PES present in the multifiber fabric.

Tween 80 and Triton X-100 often display similar characteristics in CO and PES fabrics. The literature on surfactant applications in dyeing processes is limited. Therefore, the selection of Tween 80 for further research was based on the application of non-ionic surfactants during the fermentation process. For instance, Ren et al. demonstrated that Tween 80 yielded higher production of prodigiosin when compared to Triton X-100 [16]. Moreover, various studies have highlighted the successful use of Tween 80 in enhancing the production of prodigiosin micelles during fermentation [56,57]. Surfactant selection and concentration optimization were emphasized to enhance prodigiosin dyeing, balancing color intensity and sustainability. Due to poor PES results, optimization focused on CO, identifying 0.44 mM Tween 80 as optimal for absorption and dispersion, and thus selected for further studies.

#### 3.2.2. Evaluation of Dyeing Performance in CO and PES Fabrics Under Alkaline and Neutral Prodigiosin

**Figure 7:**
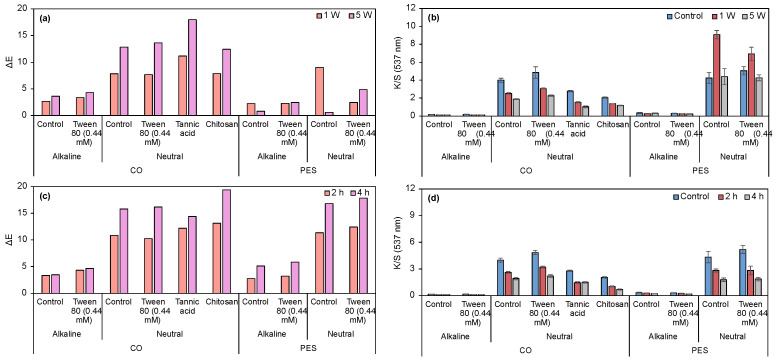
ΔE for washing (**a**) and UV light fastness (**c**) and K/S for washing (**b**) and UV light fastness (**d**) of dyed CO and PES fabrics dyed with alkaline and neutral prodigiosin batches, without and with surfactant.

Preliminary dyeing tests using the multifiber fabric (Table 2) suggest that the pH of prodigiosin extraction considerably influences its affinity toward the textiles. Samples dyed using an alkaline prodigiosin exhibited markedly lower color intensity compared to the multifiber dyed with neutral prodigiosin. The apparent colors ranged from a very pale purple to a dark shade of pink-red, respectively. The incorporation of a non-ionic surfactant into the dyeing system did not result in a perceptible ΔE. Visual assessment of CO and PES samples (Table S9, control samples) further demonstrated reduced dyeing efficiency with the alkaline batch, as evidenced by lower K/S values. In contrast, higher exhaustion of prodigiosin from the neutral batch was observed, resulting in enhanced biopigment absorption by the fabrics. The K/S and ΔE values of the dyed samples (control and washing fastness for 1 and 5 cycles) are presented in Figure 7.

K/S values of samples dyed with neutral prodigiosin batch are higher than 4, regardless of fabric composition (CO or PES). Conversely, reduced solubility of the alkaline pigment resulted in markedly lower dye absorption and K/S values, not exceeding 0.34. The non-ionic surfactant contributed to the pigment exhaustion process, where its effect was particularly evident when comparing the K/S values of CO and CO+Tween samples (neutral batch). Following washing fastness assay (1 and 5 cycles), the ΔE was analyzed. The loss of color with washing cycles is more noticeable in the substrates dyed using the neutral batch, possibly due to the higher percentage of biopigment on their surfaces. Overall, the neutral prodigiosin yielded superior results on both fabrics. Furthermore, the application of Tween 80 at 0.44 mM enhanced the K/S value when applied to neutral prodigiosin on PES fabrics. However, the improvement was not substantial in the absence of the surfactant, when compared to the acidic batch.

Non-metallic mordanting processes were evaluated on CO fabrics due to their inferior K/S compared to PES. Ch and TA were tested as biomordants to enhance the biopigment absorption and fixation, thereby improving color fastness. In addition to their application as biomordants, the biological properties of TA have been shown to positively influence the wound healing process [58]. In contrast to what was expected, CO+TA and CO+Ch samples presented lower K/S values compared to the un-mordanted sample (CO+Tween, neutral batch). These results may be due to the fact that the mordant concentration was too low (2%). ATR-FTIR analysis did not allow confirmation of chemical interaction between the CO fabric and the tested biomordants (Figure S2). Abirami et al. demonstrated that a pre-mordanted jute fabric with 10% of tannic acid increased prodigiosin dyeability, where K/S values were 27% higher when compared to the un-mordanted fabric [59]. Thus, it can be assumed that an increase in mordant concentration could increase K/S in future experiments. Furthermore, ΔE was quantified before and after light fastness tests, being higher in the dyed specimens using neutral prodigiosin. Nevertheless, replacing metallic mordants with environmentally benign natural alternatives remains essential for sustainable textile processing. For example, Zhang et al. utilized metallic mordants in CO dyeing processes with prodigiosin (in acidic conditions) [31]. It was noticed that the mordant processes using Cu^2+^ and Al^3+^ had no positive influence on color strength values. When Fe^3+^ was used as a mordant, there was a K/S improvement (~33% higher than un-mordanted dyed CO), but with major color changes, from purple to yellow. Mouro et al. studied prodigiosin multifiber fabric dyeing with FeSO_4_ and L-cysteine mordants [60]. It was noticed that using equal mordant concentrations, similar K/S values were obtained in CO fabrics. In PES fabrics, K/S values were higher (16 to 30% depending on the used concentration) using L-cysteine. Furthermore, dyeing equalization values for L-cysteine were lower than for FeSO_4_, for all fibers in the multifiber fabric, meaning that these dyed samples were more uniform in terms of color. Upon washing, metallic mordanted fabrics can release metal ions, which may end up in water beds, being prejudicial to the environment. Therefore, metallic mordants could be replaced by natural mordants, resulting in similar or superior color strength values.

Table S10 shows the ratings assigned to the specimens for color change and staining after washing. Regarding the visual assessment of color change, only CO and CO+Tween 80 samples (alkaline batch) were rated with a value lower than 3. In this case, the total leaching of the dye was observed after 5 wash cycles. The ratings obtained for color staining varied between 2/3 and 5. It should be noted that the excellent ratings of CO and CO+Tween 80 (alkaline batch) are due to the low dye concentration in the original substrates. In mordant-dyed samples, there was a slight improvement in the degree of staining when compared to the control sample (CO+Tween 80, neutral batch). For rubbing fastness, samples exhibited very good results in color change, ranging from 4 to 5. As for staining, there was a higher color transfer to adjacent undyed fabrics when the neutral prodigiosin batch was used. Again, in these samples, the amount of prodigiosin on the fabric surface is higher; thus, more biopigment can be released when the fabric is washed.

Generally, the K/S values reported in the literature are lower than those observed in this study. Ren et al. reported a maximum K/S value of 3.65 for PES fabrics functionalized at 120 °C using prodigiosin nanomicelles produced by *S. marcescens*, with Tween 80 incorporated during the fermentation process (in this study, K/S = 5.06 in PES+Tween 80 at 0.44 mM, with neutral prodigiosin) [61]. Gong et al. achieved a maximum K/S value of only 0.81 at pH = 3 for CO fabrics functionalized with prodigiosin nanomicelles (in this study, K/S = 5.38 in CO+Tween 80 at 0.008 mM, with acidic prodigiosin) [62]. Venil et al. obtained a dyeing intensity of 7.00 in CO fibers, at 60 °C and pH = 6, by applying a 5% prodigiosin solution derived from the same *S. marcescens* strain used by Ren et al. This value is comparable to the K/S value of 3.99 in CO with neutral prodigiosin obtained in the current study, although Venil et al. used a concentration approximately 40-fold higher [63]. Mouro et al. evaluated multifiber fabrics with 30 wt% biopigment produced by *Serratia plymuthica*, resulting in maximum K/S values of 0.86 (pH = 4.0) and 0.90 (pH = 8.3) for CO, and 1.10 (pH = 4.0) and 0.95 (pH = 8.3) for PES (in this study, K/S = 3.99 in CO with neutral prodigiosin and 5.40 in PES with acidic prodigiosin) [60]. Thus, through the application of diverse batches of prodigiosin and non-ionic surfactants, it is feasible to obtain K/S values that exceed those documented in the literature for biopigments produced by different bacterial strains.

**Figure 8:**
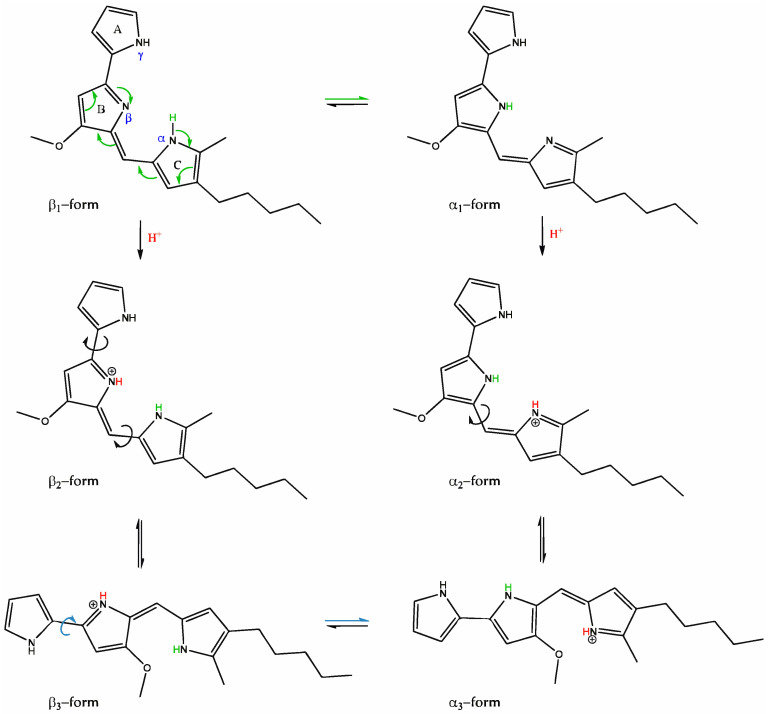
The proposed protonation mechanism of neutral prodigiosin (α_1_ and β_1_ forms) under acidic conditions (α_3_ and β_3_ forms), where β_1_ is the most stable neutral form (adapted from [65]).

As evidenced by UV–Vis spectra at different pH values, prodigiosin exhibits three distinct absorption bands, indicating its three different protonation states. Under neutral pH, two absorbance bands are observed, associated with the α and β chemical equivalence structures (α1 and β1 forms in Figure 8), being in accordance with the reported pKa of prodigiosin, encompassing between 7.2 and 7.9 [27,35,64]. In acidic conditions, protonation of both α and β pyrroles occurs, leading to a near-complete overlap of the 500 nm absorbance band with that at 537 nm, with a small shoulder being noticeable in the 500 nm region. After protonation, prodigiosin is converted into a more stable rotamer for both α and β forms (Figure 8). This rotamer conversion can possibly justify the remaining small shoulder, as the most stable rotamer will be presented in a higher percentage than the other [48,65]. On the other hand, in alkaline conditions, a hypsochromic shift to 469 nm is observed, indicating that the deprotonation of at least one nitrogen atom of the tripyrrole system will occur [22,35]. The γ-pyrrole remains neutral in acidic conditions, as it cannot be conjugated with the β-N atom to form an isomeric conformer; otherwise, β-pyrrole loses its aromaticity (high energies required). Thus, γ-NH will have an acidic behavior, and it will be the first (if not the only) one to be deprotonated, being negatively charged. Deprotonation of prodigiosin’s pyrrole groups is often omitted in the literature, as most studies only report protonated structures and the respective results, for both experimental work and theoretical calculations. The authors suggest that in future studies, the deprotonation mechanism of prodigiosin should be assessed (possibly with 1H-NMR and 15N-NMR), in order to fully comprehend its halochromic behavior.

Hence, since prodigiosin binding to fabrics will be done by hydrogen bonds, a neutral prodigiosin will have these interactions maximized, with all three N atoms available for hydrogen bond formation, in opposition to a charged prodigiosin (higher electrostatic repulsion). In contrast, cationic prodigiosin will form a lower number of hydrogen bonds due to pyrrole protonation, impairing the charged N atom from forming a bond with the fabric, where only two -NH groups will be available to form these bonds. In an anionic prodigiosin, supposing that all N atoms are deprotonated, the negative charge can be distributed between the three pyrroles, creating a strong electronegative cloud, yielding a strong repulsion against both -OH groups of CO, and the aromatic rings and ester groups of PES. If this occurs, there will be very few groups available to form hydrogen bonds and, therefore, less biopigment retained in the fabric. This proposed protonation/deprotonation mechanism is in agreement with the results achieved in this study, as fabric functionalization with alkaline prodigiosin was the weakest of the results obtained, followed by acidic prodigiosin, and neutral prodigiosin with the most promising results, as more biopigment was retained in both CO and PES fabrics. These results are consistent with the existing literature [66].

#### 3.2.3. Application of Biopigment in Conventional Printing: A Comparative Study of Commercial Thickener and Biopolymers

**Figure 9:**
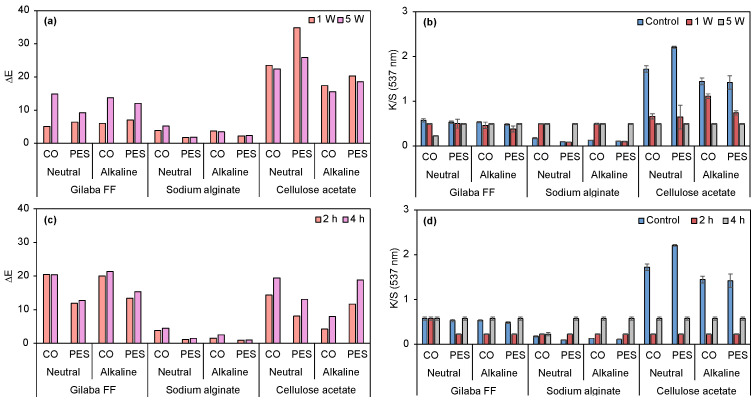
ΔE for washing (**a**) and UV light fastness (**c**) and K/S for washing (**b**) and UV light fastness (**d**) of dyed CO and PES fabrics printed with prodigiosin using alkaline/neutral batches and thickened with Gilaba FF, sodium alginate, or cellulose acetate.

Considering the obtained dyeing results, only the neutral (most effective) and alkaline (least effective) prodigiosin were selected for further investigation. Prodigiosin was selected to evaluate whether the printing technique was more suitable than dyeing, since mechanical pressure was applied, and it could help prodigiosin penetrate the fibers. For comparative purposes, neutral and alkaline prodigiosin were also combined with a commercial thickener to evaluate the performance of the biopolymers as printing thickeners. Following crosslinking with CaCl_2_, printed samples containing sodium alginate exhibited low K/S values for both pigment types and fabric substrates. Consequently, minimal ΔE was observed after washing and light fastness assays. Photographic documentation and color coordinates are presented in Table S11, and the corresponding K/S and ΔE values are shown in Figure 9.

Regarding cellulose acetate, neutral prodigiosin presented higher K/S values for both CO and PES compared to alkaline prodigiosin-functionalized fabrics. In terms of washing fastness, the ΔE differences between 1 and 5 washing cycles are not considerable, except for neutral PES fabric. The K/S values of all samples are similar after the 5 washing cycles. As for light fastness, CO printed with alkaline prodigiosin presented the lowest ΔE, followed by neutral PES, alkaline PES, and neutral CO. Overall, after 4 h of UV exposure, ΔE values increased while K/S values remained similar to the values after 2 h of exposure. To evaluate the effectiveness of these biopolymers as thickeners for printing processes, results were compared with those for samples printed with the commercial thickener Gilaba FF. ΔE increased in all tested samples, being more prominent in CO fabrics. K/S values remained similar for all samples, except for neutral CO, where K/S values were the lowest after 5 washing cycles. As for light fastness, CO samples presented higher ΔE, independently of the prodigiosin used, while PES fabric printed with neutral prodigiosin showed superior performance compared to those treated with alkaline prodigiosin.

Both Gilaba FF and cellulose acetate printed samples presented excellent rubbing fastness, independently of fiber and prodigiosin batch (Table S12). Thus, comparing the biopolymers with the commercial thickener, overall alginate yielded inferior results, and cellulose acetate demonstrated superior performance compared to the commercial thickener. Among the three thickeners, cellulose acetate produced the highest K/S values. Hence, cellulose acetate can be applied as a greener thickener alternative for sustainable printing processes, as it is a biodegradable (degradation by microorganisms in aerobic and anaerobic conditions) and non-toxic polymer [67,68]. In opposition, the commercial thickener base component is a vinyl acrylic, which, when polymerized, can be difficult to degrade naturally or even by enzymatic processes [69]. Although the change in process, from dyeing to printing, increased K/S values of alkaline prodigiosin-functionalized fabrics, these values remained inferior to those obtained with neutral prodigiosin. Furthermore, neutral prodigiosin-printed samples presented lower K/S values than dyed ones. According to these results, neutral prodigiosin presented the most promising performance in all tested application processes, probably due to the fact it can form a higher number of hydrogen bonds in neutral conditions.

#### 3.2.4. Halochromic Properties of Prodigiosin

As prodigiosin has been proven to have halochromic properties in solution, tests were performed to determine whether this property persisted following fabric functionalization. Ten alternating immersion cycles in NaOH and HCl were applied to dyed and printed samples treated with the neutral prodigiosin batch (Table 3). In dyed samples, halochromism was visible for CO, but not for PES samples, probably due to the fact that prodigiosin established more hydrogen bonds with PES. Thus, prodigiosin interaction with PES was stronger than with CO, as previously validated. Up to 5 cycles, the reversibility was immediate (less than 1 s) both for NaOH and HCl dipping. After the fifth cycle and up to the tenth, the reversibility from acid to alkaline was slower (3 to 5 s), whereas the reversibility back to acid remained immediate. This was observed for both CO samples, with and without surfactant. Color change photographs from the first and tenth cycles are presented in Table 3.

As for printed samples, halochromic behavior was performed on CA and Gilaba FF printed samples for both CO and PES. Alginate samples were not tested due to low K/S values. The fact that PES presented halochromic in printed samples, but did not in dyed samples, may be derived from the longer contact time between prodigiosin and PES fabric in dyeing, when compared to printing processes. Furthermore, in printing, prodigiosin was mixed with a printing thickener, diminishing exposure of prodigiosin to the fabric, which could compromise hydrogen bond formation. In CO+CA printed samples, reversibility from alkaline to acid was immediate, whereas from acid to alkaline color reversibility took 2 to 7 s. As for CO+Gilaba FF, reversibility times were between 3 and 6 s both for acid and alkaline dipping. Regarding PES, samples printed with CA showed immediate to 2 s reversibility from alkaline to acid, and 4 to 8 s to revert again to alkaline. Finally, PES samples printed with Gilaba FF showed a constant reversibility time of 6 s when reverting from acid to alkaline. To revert again to acid color, these samples took 2 to 4 s. Reversibility assays were also performed in washed samples (5 cycles), in which only dyed CO samples continued to show halochromic properties. The complete loss of halochromism in printed fabrics after washing indicated minimal pigment retention, likely due to low prodigiosin affinity to both fiber and thickener under printing conditions. Therefore, except for dyed PES fabrics, prodigiosin-functionalized textiles, particularly those dyed, may serve as effective pH-sensitive materials for non-washable applications such as wound dressings.

#### 3.2.5. Swelling

**Figure 10:**
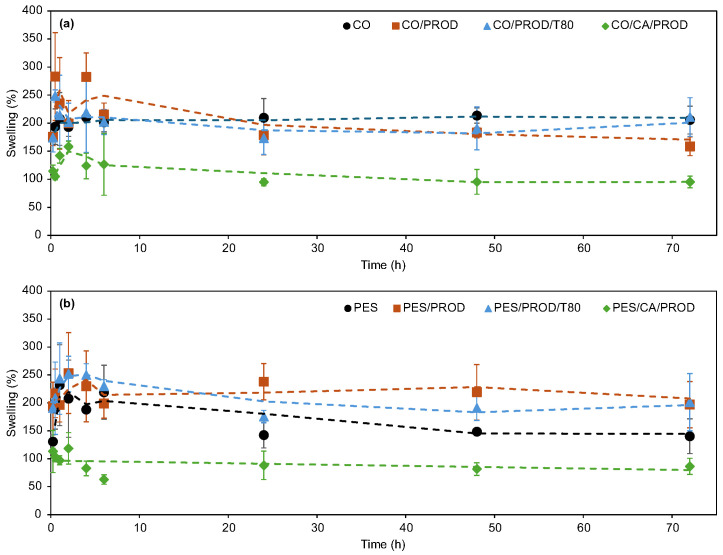
Swelling of the dyed and printed samples with neutral prodigiosin using (**a**) CO and (**b**) PES as substrate fabrics. PROD: prodigision, CA: cellulose acetate; T80: Tween 80.

The swelling capacity has been documented as an important characteristic of wound dressings. It has been associated with exudate absorption, the maintenance of a moist environment, and the reduction of skin maceration, factors considered essential for therapeutic effectiveness and patient comfort [70,71]. Accordingly, the liquid retention capacity of the optimized dyed and printed samples was evaluated by monitoring their swelling behavior in PBS over time, thereby indicating their fluid absorption capability. The corresponding results are shown in Figure 10.

The swelling capacity of the optimized samples varied depending on the substrate and functionalization process. The dyed samples exhibited swelling values ranging from 160 to 283% for CO and from 130 to 253% for PES. Overall, the lower swelling observed in PES compared to CO can be attributed to the inherently hydrophilic nature of CO fabrics [72]. The incorporation of prodigiosin and surfactant into the dyed CO samples did not lead to any substantial alteration in swelling behavior, suggesting that the liquid retention properties of the fabrics remained largely unaffected by these modifications. In contrast, a notable improvement in swelling capacity was observed in the dyed PES samples following functionalization with the biopigment. This enhancement is presumed to result from increased hydrophilicity imparted by the surface treatment. However, both CO and PES printed samples demonstrated slightly lower swelling values than their respective control or dyed samples, a phenomenon attributed to the hydrophobic nature of cellulose acetate. Nevertheless, the printed samples maintained fluid absorption levels exceeding 100%, indicating adequate liquid retention. The considerable swelling exhibited by the functionalized materials suggests their potential suitability for application in wound dressings.

#### 3.2.6. Chemical and Morphological Characterization by ATR-FTIR and SEM

**Figure 11:**
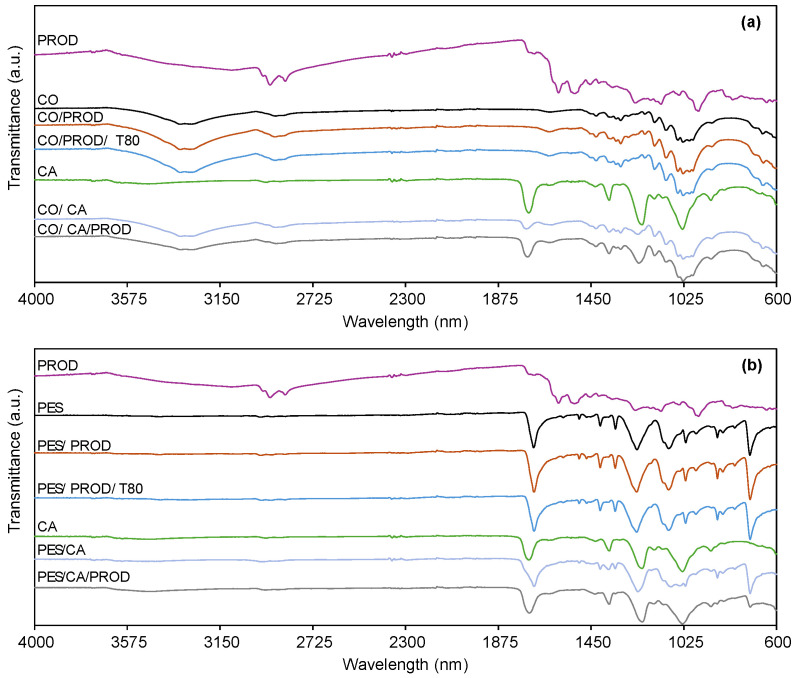
ATR-FTIR spectra of the dyed and printed samples with neutral prodigiosin using (**a**) CO and (**b**) PES as substrate fabrics. PROD: prodigision, CA: cellulose acetate; T80: Tween 80.

The FTIR absorption spectrum of isolated prodigiosin (Figure 11, gray spectra) exhibits its characteristic absorption bands, such as at 2917 cm^−1^ (asymmetric stretching of aromatic C-H groups), 2848 cm^−1^ (symmetric stretching of methylene groups or asymmetric stretching of aromatic C-H groups), and 1596 cm^−1^ (aromatic C = C and N-H bending) [49,73,74]. After incorporation into CO and PES fabrics, the absorption bands of prodigiosin are not identifiable in CO (Figure 11a, pink spectra). This is due to the overlapping characteristic bands of the respective fabric, which are higher in proportion. In the PES sample (Figure 11b, pink spectra), as prodigiosin is physically entrapped into internal polyester fibers, ATR is incapable of detecting prodigiosin peaks, since this analysis is only able to detect surface signals (0.2 µm) [6,75,76]. The strong hydrogen bond interactions and π-π stacking may also be contributing to the low detection of these peaks. These findings are consistent with the halochromic assays, in which the dyed PES did not exhibit halochromism. Conversely, cellulose acetate is easily identified only in the CO fabrics (Figure 11a, orange and light blue spectra) by its distinctive bands at 1733 cm^−1^ (O = C-O stretching), 1368 cm^−1^ (C-H vibration), and 1198 cm^−1^ (C-O stretching) [77,78]. To overcome these FTIR limitations regarding peaks of prodigiosin, SEM and SEM-EDS were performed to confirm prodigiosin’s presence in dyed and printed fabrics.

**Figure 12:**
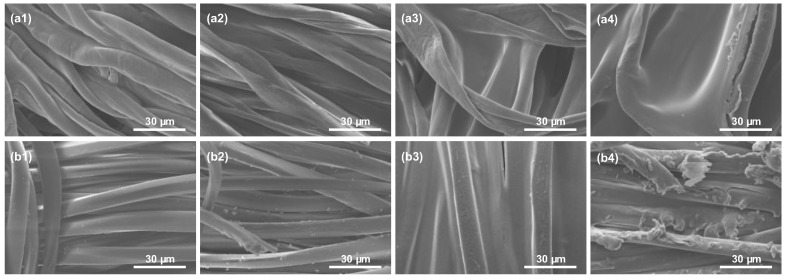
SEM images of CO (**row a**) and PES (**row b**) fabrics: (**a1**,**b1**) untreated; (**a2**,**b2**) dyed with neutral prodigiosin and Tween 80; (**a3**,**b3**) printed with cellulose acetate, and (**a4**,**b4**) printed with cellulose acetate, neutral prodigiosin, and Tween 80.

The presence of prodigiosin was not confirmed through SEM analysis in either the dyed or printed samples (Figure 12), and no morphological differences were identified between the control and treated groups. These results are consistent with those previously reported in the literature for fabrics and composites [31,60,79]. Therefore, elemental composition was performed through SEM-EDS analysis. Nitrogen signals were not observed in the elemental spectra (Table 4), with only carbon and oxygen being detected, elements commonly present in textiles, cellulose acetate, and prodigiosin. This absence of nitrogen detection is attributed to the low concentration of the biopigment within the fibers, which is below the detection limit of the applied technique (1000 ppm/0.1 wt%) [80,81]. Moreover, the limited analytical sensitivity of ATR-FTIR, SEM, and SEM-EDS techniques hindered the identification of possible interactions between the NH groups of the biopigment and the functional groups present in the textile substrates. Despite the limitations of the analytical techniques, the distinct pink coloration observed in the functionalized fabrics provided visual evidence of the biopigment’s presence within the fiber structure, even at low concentrations.

#### 3.2.7. Evaluation of Antimicrobial Properties in Prodigiosin-Funcionalized Fabrics

The presence of pathogenic agents at the wound site can interfere with the normal healing process and contribute to the onset and progression of infection [82]. Among the most implicated bacteria in wound-related and hospital-acquired infections are *S. aureus*, *E. coli*, and *P. aeruginosa* [83,84,85]. The antimicrobial potential of prodigiosin-functionalized fabrics was evaluated against these microorganisms due to their high prevalence in hospital settings and well-established association with nosocomial infections, to explore their potential application in wound dressing.

**Figure 13:**
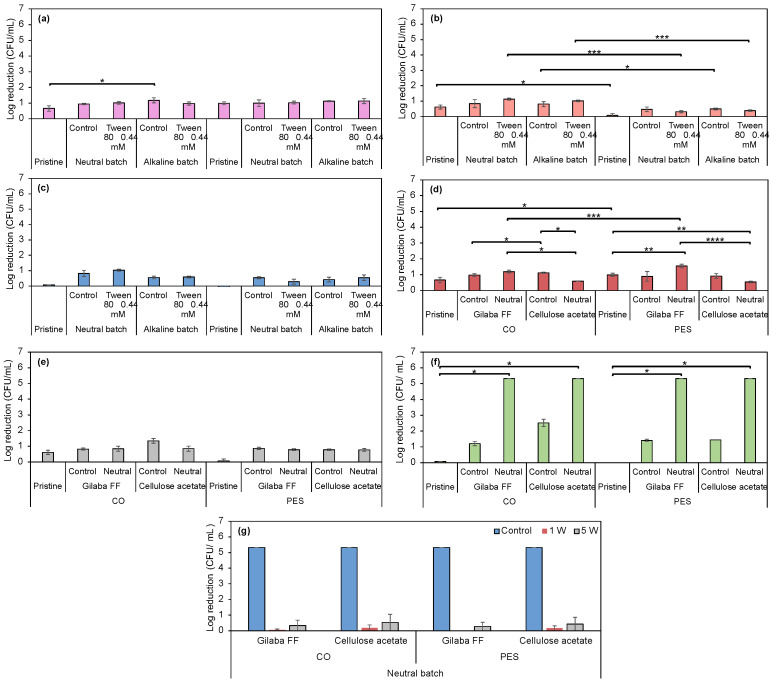
Antimicrobial results of CO and PES fabrics dyed with neutral and alkaline batches against *S. aureus* (**a**), *E. coli* (**b**), and *P. aeruginosa* (**c**), printed with Gilaba FF or cellulose acetate and neutral prodigiosin pigment against *S. aureus* (**d**), *E. coli* (**e**), and *P. aeruginosa* (**f**), and performance of the washed printed samples against *P. aeruginosa* (**g**). In panels (**a**–**f**), “Pristine” indicates unmodified CO and PES fabrics and “Control” indicates fabrics without surfactant in dyed samples, and without prodigiosin in printed samples. In panel (**c**), “1 W” and “5 W” indicate that the fabric was washed 1 or 5 times, respectively. * *p* < 0.05; ** *p* < 0.01; *** *p* < 0.001; **** *p* < 0.0001.

The antimicrobial activity was evaluated for CO and PES fabrics dyed using alkaline and neutral batches of the biopigment with an optimized concentration of Tween 80. Furthermore, CO and PES samples printed with Gilaba FF or cellulose acetate thickeners and neutral prodigiosin were also analyzed. Results are presented in Figure 13.

In general, dyed samples exhibited antimicrobial activity against the tested microorganisms, either as a decontaminant or disinfectant. Notably, the untreated CO and PES fabrics (control samples) exhibited negligible antimicrobial activity (below 1-log reduction), confirming that this property is not inherent to textile materials [86,87,88,89]. Following functionalization with neutral and alkaline batches of the biopigment, a slight enhancement in antimicrobial efficacy was detected for both fabric types against all tested microorganisms (between 0.5 and 1-log reduction). CO fabrics functionalized with alkaline prodigiosin exhibited a statistically significant difference when compared to the pristine samples against *S. aureus* (*p* < 0.05). However, the introduction of surfactants did not result in significant differences in antimicrobial activity in PES fabrics, regardless of the applied batch. Nonetheless, combining the neutral batch with 0.44 mM Tween 80 led to a slight enhancement in CO fabric antimicrobial activity against *E. coli*, *S. aureus*, and *P. aeruginosa* (*p* < 0.05), categorizing them as weak decontaminants (1 ≤ log reduction < 2) against all microorganisms [47]. Although prodigiosin has been previously reported to exhibit antimicrobial activity, its effectiveness is reduced when bound to the fabric [90]. Overall, the antimicrobial activity against *S. aureus* was considerably similar regardless of the substrate, prodigiosin batch, and treatment applied. In contrast, CO exhibited slightly superior activity against *P. aeruginosa*, with significant differences observed in the neutral batch + Tween 80 condition (*p* < 0.0001). Moreover, CO consistently demonstrated higher antimicrobial activity against *E. coli* in all tested conditions, except when treated with neutral prodigiosin biopigment, where no statistically significant difference was observed relative to PES.

Regarding the printed samples, neither Gilaba FF nor cellulose acetate exhibits antimicrobial activity. Nevertheless, when applied to both fabrics, it showed strong decontamination (5 < log reduction < 6) against *P. aeruginosa*, indicating a synergistic effect between the pigment and the applied thickener. Consequently, an analysis of the antimicrobial activity of these samples after 1 and 5 washing cycles revealed a decline in activity against *P. aeruginosa*. This highlights the necessity for implementing post-treatments to improve washing fastness and sustain antimicrobial and halochromic properties. In the dyed samples, the extended exhaustion time and the increased availability of the fiber likely contributed to the reduced antimicrobial activity, as this process facilitated deeper penetration of the biopigment into the fibers. These differences are supported by the variations in K/S values, which were considerably higher in the dyed samples for both types of fabrics with neutral prodigiosin: 3.99 and 4.24 for CO and PES dyed, respectively (Figure 7c), and 1.72 and 2.21 for CO and PES printed samples, respectively (Figure 9c). Differences in antimicrobial activity can be attributed to the amount of prodigiosin retained by the fiber [30]. Moreover, since there was a loss of activity against *P. aeruginosa* and halochromic properties, part of the biopigment was probably removed from the fabric during the washing cycles.

The antimicrobial mechanism of prodigiosin requires further analysis, as there is no consensus in the literature. A mechanism proposed for prodigiosin activity involves altering the intracellular pH of bacteria, promoted by the transport of H^+^ and Cl^−^ ions through the hydrophobic barrier of phospholipid bilayers [91]. In contrast, Ren et al. proposed that the biopigment affects RNA and protein synthesis, cellular respiration, and outer membrane disruption in Gram-negative bacteria such as *E. coli*, while affecting autolysin production and enzyme inhibition in Gram-positive bacteria like *S. aureus* [29,32,92]. The lack of activity against *E. coli* may be attributed to the inefficacy of fabric-bond prodigiosin penetration through the lipopolysaccharide external monolayer of the outer membrane of this bacterium, thus hindering its efficacy [93]. Despite reports of higher antimicrobial activity against Gram-positive bacteria [28,61,92], this property varies depending on bacterial strain, structural morphology, and compound concentration [22,63,91]. These factors likely contribute to the higher antimicrobial activity against *P. aeruginosa* observed in printed samples.

## 4. Conclusions

In this study, the highest prodigiosin yield reported for *P. putida* strains (1.85 ± 0.19 g/L) was achieved by testing a different mineral medium formulation from what is described in the literature. Even though this work did not aim at a thorough optimization of the prodigiosin production in strain PIG02, it hints at the possibility of further increasing prodigiosin yield by combining additional engineering approaches with a comprehensive physiological optimization (e.g., by using the Plackett–Burman method and central composite design), to be pursued in the following studies. Crude prodigiosin extracts were prepared under various pH conditions, which proved essential for obtaining optimal prodigiosin-functionalized fabrics. Despite applying a prodigiosin concentration (0.125%) lower than those found in the literature (0.4% or higher), promising antimicrobial and fastness results were achieved. Furthermore, non-ionic surfactants improved the prodigiosin dispersibility, thereby enhancing fabric dyeability. Among the tested conditions, the neutral prodigiosin batch exhibited superior functionalization outcomes compared to the acidic and alkaline batches. Prodigiosin-dyed cotton fabrics presented halochromic properties, potentializing their application as pH sensors for technical textiles. These halochromic responses were fully reversible and remained stable for at least 10 pH switching cycles, indicating reliable performance under repeated stimuli. Although halochromic properties were not observed in the dyed polyester fabrics, higher color strength (K/S_537nm_ = 5.19) and improved washing fastness were achieved compared to cotton fabrics (K/S_537nm_ = 4.85), thereby supporting the potential of prodigiosin to replace synthetic purple/violet dyes, enhancing the sustainability of the present work.

Cellulose acetate biopolymer proved to be a viable substitute for commercial printing thickeners. Fabrics printed with prodigiosin using cellulose acetate as a thickener presented K/S_537nm_ values of 2.21 (PES) and 1.72 (CO), exhibited halochromism, as well as excellent antimicrobial activity against *P. aeruginosa* (log reduction > 5). Moreover, the functionalized fabrics demonstrated a notable swelling capacity (>100%), an advantageous property for wound dressings, as it enhances fluid absorption and helps maintain a moist healing environment. These results support the feasibility of using such materials in biomedical applications, particularly in the development of antimicrobial and pH-responsive wound dressings.

## Figures and Tables

**Table 1:** Properties of the evaluated fabrics.

Properties/ Fabrics	Cotton	Polyester	Silk	Wool	Polyamide
Substrate	Fabric	Fabric	Fabric	Fabric	Fabric
Composition	100% CO	100% PES	100% SK	100% WO	100% PA
Fabric construction	Plaine-weave	Plaine-weave	Plaine-weave	Plaine-weave	Plaine-weave
	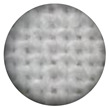	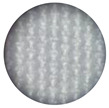	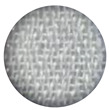	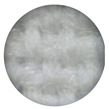	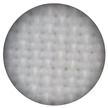
Density in yarns/cm (warp × weft)	33 × 30	32 × 26	140 × 50	16 × 14	46 × 34
Mass per unit area (g/m^2^)	137	100	57	212	115
Thickness (mm)	0.27	0.26	0.20	0.47	0.27

**Table 2:** Multifiber fabric dyed with prodigiosin alkaline/neutral batch without and with surfactant (Tween 80).

	Acidic Batch (from Table S4)		Neutral Batch		Alkaline Batch	
Multifiber Composition	Without Surfactant	Tween 80	Without Surfactant	Tween 80	Without Surfactant	Tween 80
CA	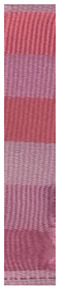	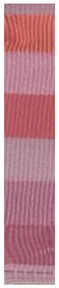	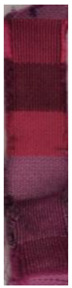	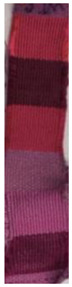	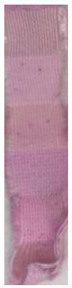	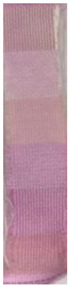
CO						
PA						
PES						
PAC						
WO						

**Table 3:** The halochromic properties and color reversibility of the fabrics functionalized by exhaustion and printing processes.

		Control (Neutral Batch)	1 Cycle		10 Cycles	
			NaOH	HCl	NaOH	HCl
Dyed samples	CO/Prodigiosin	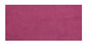	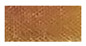	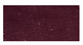	* 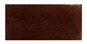 *	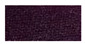
	CO/Prodigiosin/ Tween 80	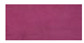	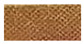	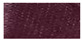	* 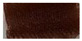 *	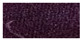
	PES/Prodigiosin	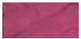	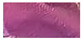	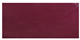	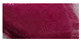	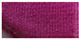
	PES/Prodigiosin/ Tween 80	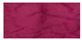	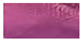	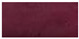	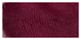	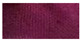
Printed samples	CO/Cellulose acetate/ Prodigiosin	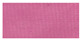	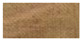	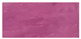	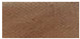	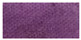
	CO/Gilaba FF/ Prodigiosin	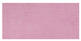	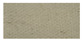	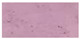	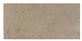	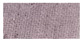
	PES/Cellulose acetate/ Prodigiosin	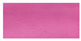	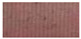	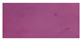	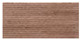	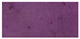
	PES/Gilaba FF/ Prodigiosin	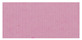	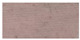	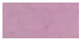	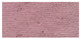	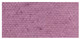

**Table 4:** SEM-EDS of the dyed and printed CO and PES samples with neutral prodigiosin.

		Elemental Composition (Mass, %)	
		O	C
Control	CO	54.76	45.24
	PES	59.88	40.12
Dyed samples	CO/Prodigiosin/Tween 80	54.48	45.52
	PES/Prodigiosin/Tween 80	58.99	41.01
Printed samples	CO/Cellulose acetate	54.13	45.87
	CO/Cellulose acetate/Prodigiosin	53.98	46.02
	PES/Cellulose acetate	53.89	46.11
	PES/Cellulose acetate/Prodigiosin	55.18	44.82

## Data Availability

Data will be made available on request.

## Supplementary Materials

The following supporting information can be downloaded at: https://www.mdpi.com/article/10.3390/biom15081113/s1, Table S1: Results of samples after dyeing, washed with distilled water and an aqueous solution with ECE [44,48]; Table S2: Initial and after exhaustion process solutions with acidic prodigiosin [63,92]; Table S3: Results of the washing fastness assay of PES, PA, and WO fabrics dyed with acidic prodigiosin [30,90]; Figure S1: The initial dyeing solutions (in dH_2_O), following the incorporation of surfactants as emulsifying agents and the application of Ultra-Turrax; Table S4: Results of the washing fastness assay of multifiber samples treated with the various surfactants and acidic prodigiosin; Table S5: Color coordinates and sample pictures of CO and PES fabrics dyed with acidic prodigiosin; Table S6: Color change and staining of washing (1 and 5 cycles), rubbing (wet and dry) and UV light (4 h) fastness of the CO and PES fabrics dyed with acid prodigiosin; Table S7: Color coordinates and sample photographs of CO and PES fabrics dyed with acidic prodigiosin and optimized with non-ionic surfactants; Table S8: Color change and staining of washing (1 and 5 cycles), rubbing (wet and dry) and UV light (4 h) fastness of the CO and PES fabrics dyed with acid prodigiosin; Table S9: Color coordinates of CO and PES fabrics dyed with prodigiosin alkaline/neutral batch without and with surfactant (0.44 mM Tween 80); pre-mordanting with TA and post-mordanting with Ch of CO fabrics dyed with neutral batch; Figure S2: ATR-FTIR spectra of CO samples pre-treated with TA or Ch and dyed with the neutral batch; Table S10: Color change and staining of washing (1 and 5 cycles), rubbing (wet and dry) and UV light (4 h) fastness of the CO and PES fabrics dyed with prodigiosin alkaline batch without and with surfactant; dyed with neutral batch without and with surfactant; Table S11: Color coordinates of CO and PES fabrics printed with prodigiosin using alkaline/neutral batches and thickened with Gilaba FF, sodium alginate or cellulose acetate; Table S12: Color change and staining of washing (1 and 5 cycles), rubbing (wet and dry) and UV light (4 h) fastness of the CO and PES fabrics printed with prodigiosin using alkaline/neutral batches and thickened with Gilaba FF, sodium alginate or cellulose acetate.

## Abbreviations

The following abbreviations are used in this manuscript:

1 W	One washing cycle
5 W	Five washing cycle
BC	Alkylbenzyldimethylammonium
CA	Acetate
CFU	Colony-forming unit
Ch	Chitosan
CIELab*	Expression of color determined by the International Commission on Illumination
CMC	Critical micellar concentration
CO	Cotton
CTAB	Cetyltrimethylammonium bromide
*E. coli*	*Escherichia coli* bacteria
FTIR	Fourier transform infrared spectroscopy
*g*	Centrifugal force
HPLC-DAD-MS	High-Performance Liquid Chromatography with Diode Array Detectors Coupled to Mass Spectrometry
IPTG	Isopropyl β-D-1-thiogalactopyranoside
K	Absorption coefficient
K/S	Color strength
Log_10_ reduction	Logarithmic reduction
MM-G	Mineral salts medium supplemented with glycerol and lactid acid
OD	Optical density
*P. aeruginosa*	*Pseudomonas aeruginosa* bacteria
*P. putida*	*Pseudomonas putida* bacteria
PA	Polyamide
PAC	Acrylic
PES	Polyester
PROD	Prodigiosin
R	Reflectance decimal fraction
RT	Retention time
S	Scattering coefficient
*S. aureus*	*Staphylococcus aureus* bacteria
SDS	Sodium dodecyl sulfate
SEM	Scanning Electron Microscopy
SEM-EDS	Energy-Dispersive Spectroscopy
SK	Silk
TA	Tannic acid
TB-G	Terrific broth supplemented with glycerol
TSA	Tryptic soy agar
TSB	Tryptic soy broth
UV	Ultraviolet
UV/Vis	Ultraviolet/visible
WO	Wool
Δa*	Red-green color difference
Δb*	Yellow-blue color difference
ΔE	Color difference
ΔL*	Lightness difference
