# Supplementary material for: Application of Prodigiosin Extracts in Textile Dyeing and Novel Printing Processes for Halochromic and Antimicrobial Wound Dressings
Source: Biomolecules. 2025 Aug 1;15(8):1113. doi: 10.3390/biom15081113 (PMC12383654; doi:10.3390/biom15081113)
Supplement: Supplementary file 1 [file biomolecules-15-01113-s001.zip › biomolecules-3737321-supplementary.pdf]

Article

Application of Prodigiosin Extracts in Textile Dyeing and Novel Printing Processes for Halochromic and Antimicrobial Wound Dressings

Cátia Alves [1,†], Pedro Soares-Castro [2,†], Rui D. V. Fernandes [1], Adriana Pereira [1], Rui Rodrigues [1], Ana Rita Fonseca [2], Nuno C. Santos [2,3], and Andrea Zille [1,*]

[1] Centre for Textile Science and Technology (2C2T), Department of Textile Engineering, University of Minho, Campus of Azurém, 4800-058 Guimarães, Portugal
[2] GIMM – Gulbenkian Institute for Molecular Medicine, Av. Prof. Egas Moniz, 1649-035 Lisbon, Portugal
[3] Faculdade de Medicina, Universidade de Lisboa, Av. Prof. Egas Moniz, 1649-028 Lisbon, Portugal.
[†] Authors contributed equally to the manuscript.
[*] Correspondence: nsantos@medicina.ulisboa.pt; azille@2c2t.uminho.pt

Supporting Information

Table S1. Results of samples after dyeing, washed with distilled water and an aqueous solution with ECE……………….3

Table S2. Initial and after exhaustion process solutions with acidic prodigiosin……………………………………………..3

Table S3. Results of the washing fastness assay of PES, PA, and WO fabrics dyed with acidic prodigiosin…………………4

Figure S1. The initial dyeing solutions (in dH_2O), following the incorporation of surfactants as emulsifying agents and the application of Ultra-Turrax: (a) without surfactant, (b) Triton X-100, (c) Tween 80, (d) SDS, (e) Lutensit, (f) CTAB, and (g) BC, with acidic prodigiosin……………………………………………………………...…………………………………………….5

Table S4. Results of the washing fastness assay of multifiber samples treated with the various surfactants and acidic prodigiosin…….………………………………………………..………………………………………………………………...5

Table S5. Color coordinates and sample pictures of CO and PES fabrics dyed with acidic prodigiosin…………………..….6

Table S6. Color change and staining of washing (1 and 5 cycles), rubbing (wet and dry) and UV light (4 h) fastness of the CO and PES fabrics dyed with acid prodigiosin………………………………………………………………………………….7

Table S7. Color coordinates and sample photographs of CO and PES fabrics dyed with acidic prodigiosin and optimized with non-ionic surfactants.…………………………………………………………... ……………………………………………8

Table S8. Color change and staining of washing (1 and 5 cycles), rubbing (wet and dry) and UV light (4 h) fastness of the CO and PES fabrics dyed with acid prodigiosin.…………………………………………………………………………………9

Table S9. Color coordinates of CO and PES fabrics dyed with prodigiosin alkaline/neutral batch without and with surfactant (0.44 mM Tween 80); pre-mordanting with TA and post-mordanting with Ch of CO fabrics dyed with neutral batch……..………..10

Figure S2. ATR-FTIR spectra of CO samples pre-treated with TA or Ch and dyed with the neutral batch…………………11

Table S10. Color change and staining of washing (1 and 5 cycles), rubbing (wet and dry) and UV light (4 h) fastness of the CO and PES fabrics dyed with prodigiosin alkaline batch without and with surfactant; dyed with neutral batch without and with surfactant………………………. ……………………………………………………………………………………12

Table S11. Color coordinates of CO and PES fabrics printed with prodigiosin using alkaline/neutral batches and thickened with Gilaba FF, sodium alginate or cellulose acetate.…………………………………………… ………………………………..13
Table S12. Color change and staining of washing (1 and 5 cycles), rubbing (wet and dry) and UV light (4 h) fastness of the CO and PES fabrics printed with prodigiosin using alkaline/neutral batches and thickened with Gilaba FF, sodium alginate or cellulose acetate……………………………………..……………….………………………………………………………..14

- ***Application of Prodigiosin and Optimization of the Solvent-free Dyeing Process***

The solubility of the biopigment was studied at an early development phase. Multifiber samples were dyed with the biopigment using several ethanol-based systems, due to their insolubility in water (intracellular pigments) [1]. Multifiber fabrics were used to select fibers with the highest affinity for the dye. Silk (SK) fabrics were specifically applied in acid conditions for their amino and carboxyl groups, which enhance dye affinity and fixation through electrostatic interactions or covalent bonds [2]. After assessing their washing fastness by mimicking a domestic washing step, staining was visible in all samples (Table S1), due to the insolubilized pigment and low dispersion in the solution. In general, the simplest application condition, ethanol:water 1:1, seems to be sufficient for dyeing most of the analyzed fibers, with the exception of SK and polyamide (PA). In the case of polyester (PES) and wool (WO), as the samples had different shades, two application conditions were selected: ethanol:water and ethanol:buffer at pH 9, both 1:1.

Table S1. Results of samples after dyeing, washed with distilled water and an aqueous solution with ECE.

		**Substrate**				
		Multifiber	Multifiber	Multifiber	Multifiber	SK
	Solvents / Multifiber	EtOH: dH_2O	EtOH: acetate buffer	EtOH: phosphate buffer	EtOH: Tris-HCl	EtOH: acetate buffer
**After dyeing process**	Acetate (CA)<br>Cotton (CO)<br>Polyamide (PA)<br>Polyester (PES)<br>Acrylic (PAC)<br>Wool (WO)	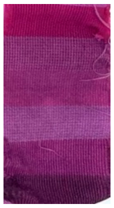	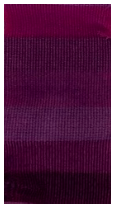	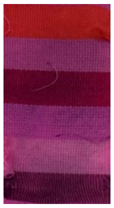	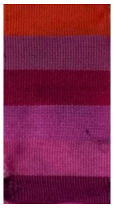	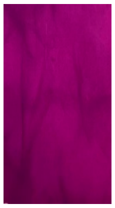
**After washing with distilled**	CA<br>CO<br>PA<br>PES<br>PAC<br>WO	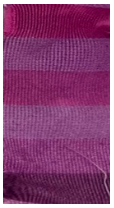	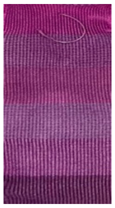	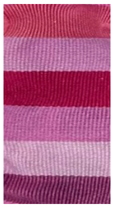	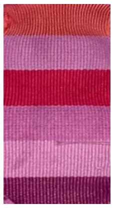	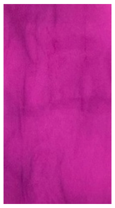
**After washing with ECE**	CA<br>CO<br>PA<br>PES<br>PAC<br>WO	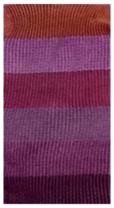	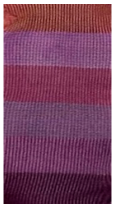	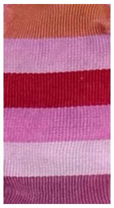	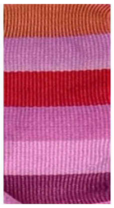	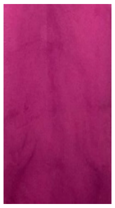

Table S2 shows the solutions after the dyeing process, where there are some considerable differences between the initial and after-dyeing solutions. Comparing the multifiber fabrics, it is evident that an increase in pH value resulted in a lower exhaustion rate. This increased the anionic character of the textile, thereby enhancing the repulsive interactions between the pigment and the fabric [3]. A near-complete exhaustion was obtained for the SK fabric dyed at an acidic pH. Ren *et al.* dyed a SK fabric with prodigiosin at 80 °C, evaluating various acidic pH conditions, and achieving a maximum exhaustion rate of approximately 67% (pH 2.1) [4]. The same authors report that the solubility of prodigiosin increases in more acidic pH conditions. This is due to the protonation of -NH groups in an acidic medium, which generates a positive charge in prodigiosin's structure, thereby improving solubility and, consequently, affinity. As a result, the exhaustion process was more effective in SK fabric under acidic pH conditions.

Table S2. Initial and after exhaustion process solutions with acidic prodigiosin.

**Substrate**	Multifiber	Multifiber	Multifiber	Multifiber	SK fabric
**Solvent (1:1)**	Ethanol: dH_2O	Ethanol: acetate buffer	Ethanol: phosphate buffer	Ethanol: tris-HCl	Ethanol: acetate buffer
**pH**	5.76	5.26	8.28	8.61	4.75
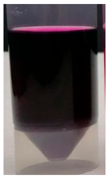	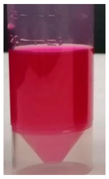	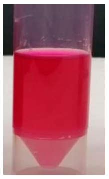	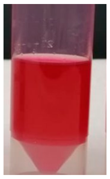	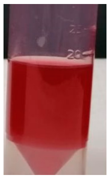	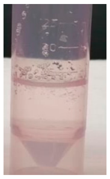
Initial	After exhaustion process				

Considering this preliminary assay, PES, WO, and PA fabrics (Table S3) were selected to carry out a scale-up using the optimal dyeing conditions identified during the preliminary assay. The study evaluated the fabrics' washing fastness properties as part of this scale-up process. According to the literature, prodigiosin exhibited a high affinity for fibers containing carbonyl and amide groups, which promote a higher hydrogen bonding than the hydroxyl groups in cellulose [5]. Consequently, PES, PA, and WO fibers demonstrated greater color intensity [6] when compared to CO (Table S3). Janković *et al.* mentions that PA fibers have amine groups only at the terminal groups, whereas WO fibers possess amine groups along the side chains and at the terminals. Thus, these structural differences lead WO and PES fibers to exhibit a higher color intensity, when compared to PA fibers. In general, dyed substrates presented bright hues and promising washing fastness results.

Table S3. Results of the washing fastness assay of PES, PA, and WO fabrics dyed with acidic prodigiosin.

**Solvents**	**PES**		**PA**		**WO**	
	Control	Washing fastness	Control	Washing fastness	Control	Washing fastness
Ethanol	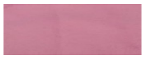	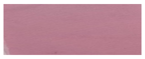	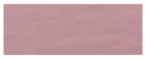	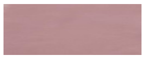	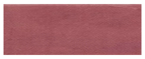	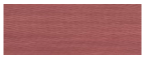
Ethanol:dH_2O	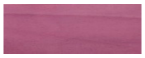	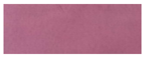	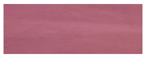	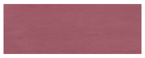	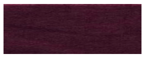	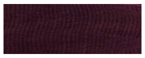
Ethanol:acetate buffer	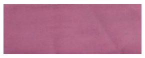	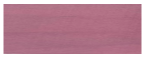	–	–	–	–
Ethanol:phosphate buffer	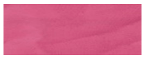	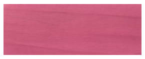	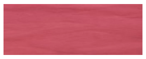	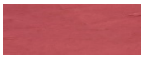	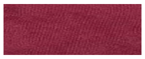	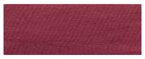
Ethanol:Tris-HCl buffer	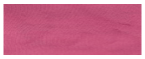	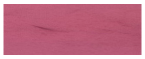	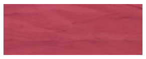	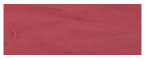	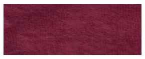	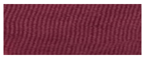

Although the dyeing of WO shows intense colors, the ethanol:water solvent system is not viable for industrial scale-up due to the volatility and flammability of ethanol. Therefore, three types of surfactants were chosen and studied as dispersing agents, to increase prodigiosin solubility and replace the ethanol:water system: non-ionic (Triton X-100 and Tween 80), anionic (Lutensit and SDS), and cationic (CTAB and BC). These surfactants were intended to function as dispersing agents, thereby enhancing the solubility of the biopigment (Figure S1).

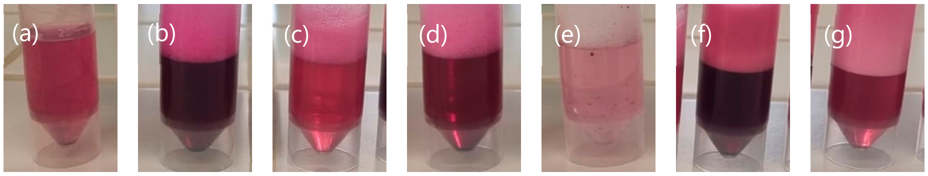

Figure S1. The initial dyeing solutions (in dH_2O), following the incorporation of surfactants as emulsifying agents and the application of Ultra-Turrax: (a) without surfactant, (b) Triton X-100, (c) Tween 80, (d) SDS, (e) Lutensit, (f) CTAB, and (g) BC, with acidic prodigiosin.

The samples from the anionic surfactant class were stained, even after washing (Table S4). In addition, different colors were observed in the samples depending on the type of fiber subjected to dyeing. Triton X-100 showed the most promising results after washing. Hence, moving forward from these results, non-ionic and cationic surfactants were selected to continue the study in CO and PES fabrics, to confirm these pre-screening results.

Table S4. Results of the washing fastness assay of multifiber samples treated with the various surfactants and acidic prodigiosin.

		**dH_2O**	**Non-ionic**		**Anionic**		**Cationic**	
			Triton X-100	Tween 80	SDS	Lutensit	CTAB	BC
**Control**	CA	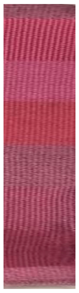	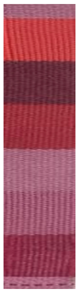	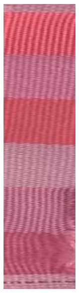	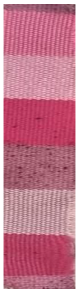	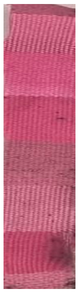	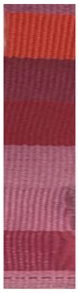	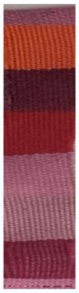
	CO							
	PA							
	PES							
	PAC							
	WO							
**Washing fastness**	CA	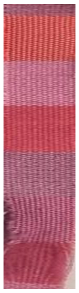	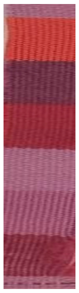	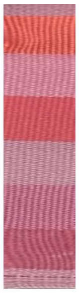	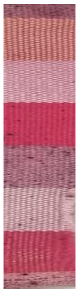	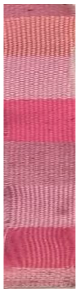	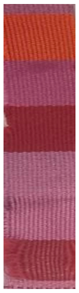	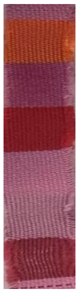
	CO							
	PA							
	PES							
	PAC							
	WO							

- ***Evaluation of Surfactants Effect on Prodigiosin Solubility and CO and PES Dyeing Process under Acidic Biopigment***

Table S5. Color coordinates and sample pictures of CO and PES fabrics dyed with acidic prodigiosin.

			**CO Fabric**				**PES Fabric**			
			**L***	**a***	**b***	**Sample**	**L***	**a***	**b***	**Sample**
**dH_2O**	Control		63.29	24.64	-7.27	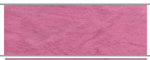	45.87	25.75	-4.57	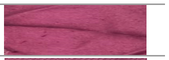
	Washing fastness	1 W	70.29	20.90	-7.86	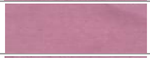	47.80	27.20	-4.02	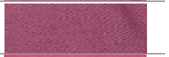
		5 W	70.19	17.08	-6.28	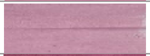	48.70	28.42	-4.11	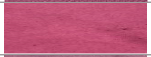
**Triton X-100**	Control		45.44	22.83	-5.48	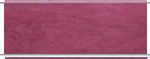	48.72	14.50	-2.11	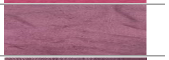
	Washing fastness	1 W	48.84	18.96	-6.33	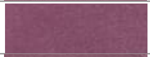	47.33	13.99	-1.56	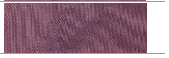
		5 W	51.58	16.12	-5.03	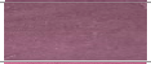	50.24	22.06	-3.15	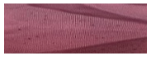
**Tween 80**	Control		55.92	25.78	-7.29	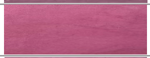	45.03	19.79	-3.54	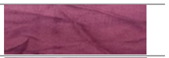
	Washing fastness	1 W	62.52	21.86	-8.77	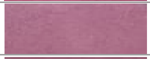	45.35	19.56	-3.40	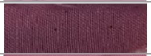
		5 W	62.10	19.13	-7.69	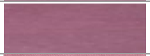	50.81	15.09	-1.22	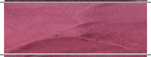
**CTAB**	Control		62.80	16.80	-1.91	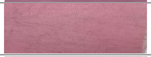	44.77	22.33	-3.36	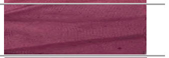
	Washing fastness	1 W	67.84	12.11	-3.04	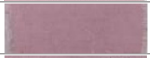	45.28	17.74	-1.04	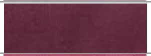
		5 W	69.70	12.23	-2.35	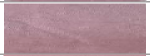	51.40	19.34	-1.27	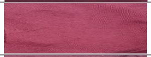
**BC**	Control		67.59	12.39	-0.71	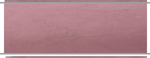	46.65	16.42	-1.11	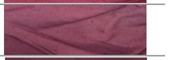
	Washing fastness	1 W	72.32	9.05	-1.62	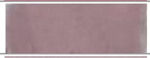	46.43	24.08	-3.71	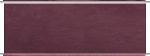
		5 W	73.97	9.00	-0.70	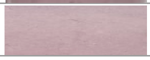	51.39	25.34	-3.57	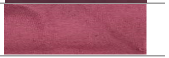

Table S6. Color change and staining of washing (1 and 5 cycles), rubbing (wet and dry) and UV light (4 h) fastness of the CO and PES fabrics dyed with acid prodigiosin.

Samples		Washing fastness														Rubbing fastness				Light fastness (4 h)
		Colour change		Staining												Colour change		Staining		
				CA		CO		PA		PES		PAC		WO						
		1 W	5 W	1 W	5 W	1 W	5 W	1 W	5 W	1 W	5 W	1 W	5 W	1 W	5 W	Dry	Wet	Dry	Wet	
CO	dH2O	3	1	3/4	5	4/5	5	2/3	4	4/5	5	4	5	4	5	5	4/5	5	5	2
	Triton	3	2	2/3	5	3/4	5	2	3/4	4	5	3/4	5	3	5	4	3/4	5	5	4
	Tween	3	2	3/4	5	4/5	5	3	3/4	4/5	4/5	4	5	4	5	4/5	4v5	5	4	3
	CTAB	2	2	3/4	5	4/5	5	3	4/5	4/5	5	4	5	4	5	5	5	5	5	2
	BC	1	2	2/4	5	4/5	5	3	4/5	4/5	5	4	5	4	5	5	4/5	5	5	1
PES	dH2O	4	4	4	5	4/5	5	3	4/5	4	5	4/5	5	4	5	5	5	4	4	4
	Triton	4	4	4/5	5	4/5	5	3/4	4	4	4/5	4/5	5	4	5	5	5	5	5	1
	Tween	4	3	5	5	5	5	4	4/5	5	5	5	5	5	5	5	5	5	5	4
	CTAB	4	3	4	5	4	5	3	5	4	5	4/5	5	4	5	5	5	5	5	4
	BC	4	4	5	5	5	5	4	5	5	5	5	5	5	5	5	5	5	5	4

Table S7. Color coordinates and sample photographs of CO and PES fabrics dyed with acidic prodigiosin and optimized with non-ionic surfactants.

				CO Fabric				PES Fabric			
				**L***	**a***	**b***	**Sample**	**L***	**a***	**b***	**Sample**
**dH2O**		Control		56.62	34.55	-9.38	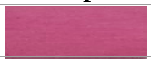	45.87	25.75	-4.57	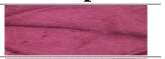
		Washing fastness	1 W	59.15	26.03	-9,74	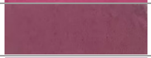	47.80	27.20	-4.02	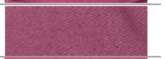
			5 W	62.16	19.73	-6.65	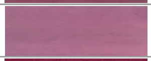	48.70	28.42	-4.11	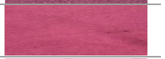
**Triton X-100**	**0.36 mM**	Control		44.51	34.43	-8.88	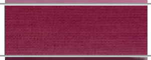	62.95	24.96	-4.21	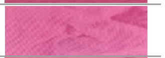
		Washing fastness	1 W	50.42	25.21	-7.52	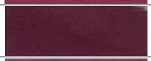	61.27	26.03	-3.44	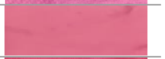
			5 W	52.67	19.56	-6.89	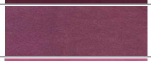	66.15	26.87	-3.47	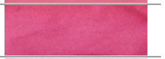
	**1.44 mM**	Control		55.55	29.18	-8.53	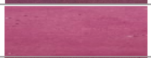	61.91	22.05	-3.22	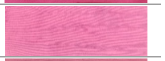
		Washing fastness	1 W	58.22	22.56	-8.12	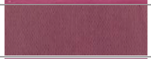	62.56	21.91	-2.65	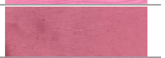
			5 W	62.49	16.88	-5.87	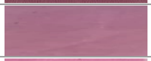	64.60	23.70	-2.78	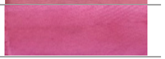
	**1.98 mM**	Control		65.24	29.54	-9.76	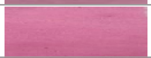	-	-	-	-
		Washing fastness	1 W	65.89	21.61	-8.36	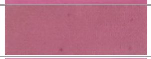	-	-	-	-
			5 W	68.86	15.19	-5.81	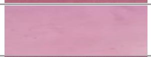	-	-	-	-
**Tween 80**	**0.008 mM**	Control		46.66	35.27	-9.77	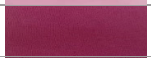	58.57	25.67	-5.19	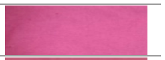
		Washing fastness	1 W	52.98	24.84	-8.14	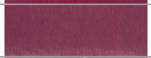	57.65	26.40	-4.29	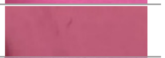
			5 W	54.48	19.81	-7.25	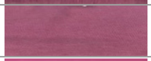	60.44	27.41	-4.51	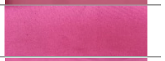
	**0.32 mM**	Control		56.30	33.37	-9.29	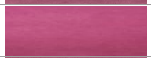	61.60	24.37	-4.11	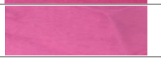
		Washing fastness	1 W	60.17	25.49	-8.99	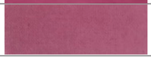	63.01	25.27	-3.53	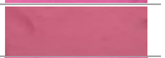
			5 W	62.84	18.02	-4.97	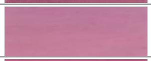	64.93	25.96	-3.56	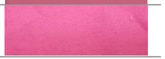
	**0.44 mM**	Control		60.02	31.11	-8.94	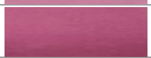	-	-	-	-
		Washing fastness	1 W	62.59	24.13	-9.07	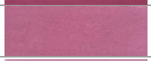	-	-	-	-
			5 W	64.02	17.92	-6.13	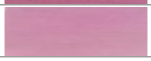	-	-	-	-

Table S8. Color change and staining of washing (1 and 5 cycles), rubbing (wet and dry) and UV light (4 h) fastness of the CO and PES fabrics dyed with acid prodigiosin.

Samples			Washing fastness														Light fastness (4 h)
			Colour change		Staining												
					CA		CO		PA		PES		PAC		WO		
			1 W	5 W	1 W	5 W	1 W	5 W	1 W	5 W	1 W	5 W	1 W	5 W	1 W	5 W	
**CO**	**dH_2O**		4	2	3/4	5	4/5	5	3/4	5	1/2	4/5	4/5	5	3/4	5	2
	**Triton X-100**	**0.36 mM**	4	3	3	5	4/5	5	2/3	5	1/2	4	3/4	5	2/3	5	3
		**1.44 mM**	4	2	3/4	5	4/5	5	3/4	5	1/2	4/5	4/5	5	3/4	5	2
		**1.98 mM**	3	2	4/5	5	5	5	4/5	5	3/4	4/5	5	5	4/5	5	2
	**Tween 80**	**0.008 mM**	4	3	3	5	4/5	5	2/3	5	172	4/5	374	5	2/3	5	2
		**0.32 mM**	3	2	3	5	4/5	5	3	5	1	4/5	4	5	3	5	2
		**0.44 mM**	4	2	4	5	4/5	5	4	5	3	4/5	4/5	5	4/5	5	2
**PES**	**dH_2O**		4	4	4	5	4/5	5	3	4/5	4	5	4/5	5	4	5	4
	**Triton X-100**	**0.36 mM**	5	4	5	5	5	5	5	5	4	4/5	5	5	4/5	5	3
		**1.44 mM**	5	4	5	5	5	5	5	5	4/5	5	5	5	5	5	4
	**Tween 80**	**0.008 mM**	5	4	5	5	5	5	5	5	4/5	5	5	5	5	5	2
		**0.32 mM**	5	3	5	5	5	5	5	5	5	5	5	5	5	5	2

- ***Evaluation of Dyeing Performance in CO and PES Fabrics under Alkaline and Neutral Prodigiosin***

Table S9. Color coordinates of CO and PES fabrics dyed with prodigiosin alkaline/neutral batch without and with surfactant (0.44 mM Tween 80); pre-mordanting with TA and post-mordanting with Ch of CO fabrics dyed with neutral batch.

			Alkaline batch				Neutral batch			
			L*	a*	b*	Sample	L*	a*	b*	Sample
**CO**	Control		80.97	2.50	-1.31	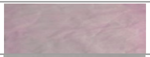	49.09	31.93	-9.13	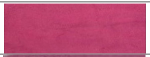
	Washing fastness	1 W	82.21	0.35	-0.32	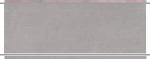	53.50	25.45	-8.76	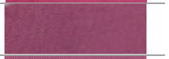
		5 W	83.38	-0.05	-0.35	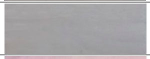	56.39	21.42	-8.16	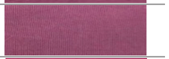
**CO+ Tween 80 (0.44 mM)**	Control		80.70	3.32	-1.63	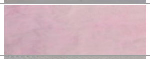	46.40	31.65	-8.34	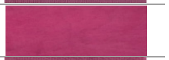
	Washing fastness	1 W	82.33	0.62	-0.47	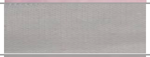	50.76	25.36	-8.98	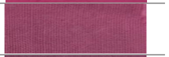
		5 W	83.14	0.01	-0.33	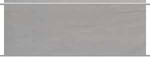	53.15	19.94	-6.44	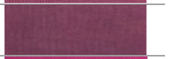
**CO+ TA**	Control		-	-	-	-	56.31	34.87	-10.30	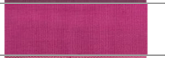
	Washing fastness	1 W	-	-	-	-	62.21	25.63	-8.18	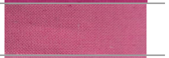
		5 W	-	-	-	-	65.95	20.03	-6.93	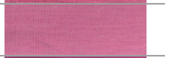
**CO+ Ch**	Control		-	-	-	-	59.47	29.51	-10.36	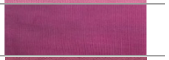
	Washing fastness	1 W	-	-	-	-	63.27	22.99	-8.16	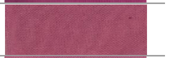
		5 W	-	-	-	-	63.75	18.73	-5.88	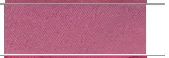
**PES**	Control		74.40	5.78	1.96	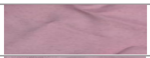	50.05	26.71	-3.68	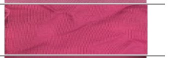
	Washing fastness	1 W	76.53	5.05	1.88	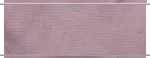	41.38	24.18	-3.41	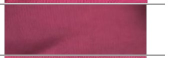
		5 W	75.18	5.54	1.98	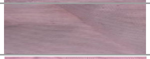	49.98	27.19	-3.32	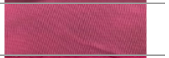
**PES+ Tween 80 (0.44 mM)**	Control		76.14	6.32	0.13	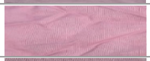	48.28	28.27	-4.14	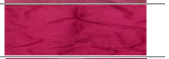
	Washing fastness	1 W	78.32	5.67	-0.23	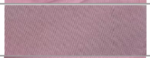	45.82	28.04	-4.06	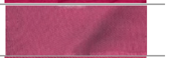
		5 W	78.26	5.06	0.27	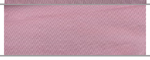	52.20	31.13	-4.51	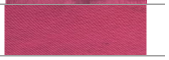

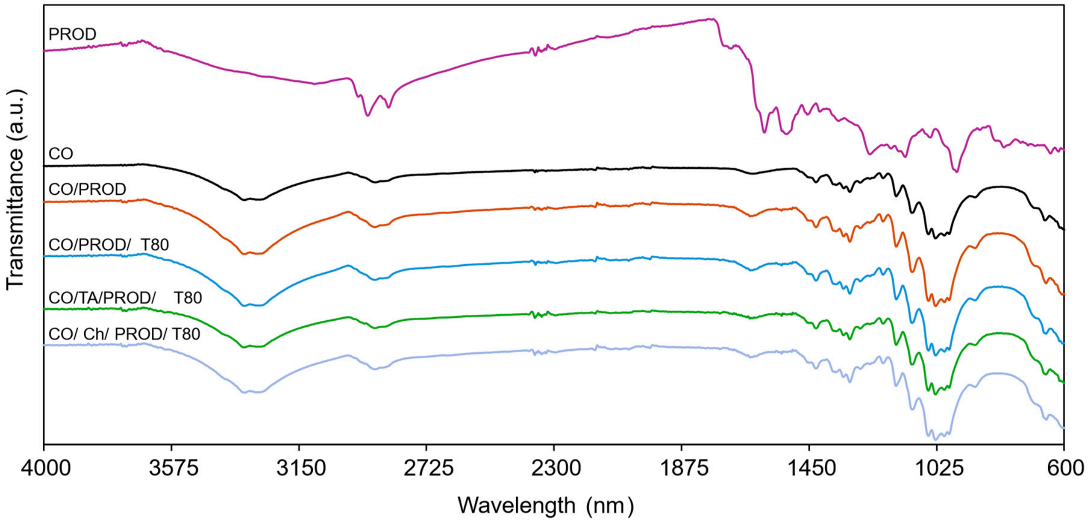

Figure S2. ATR-FTIR spectra of CO samples pre-treated with TA or Ch and dyed with the neutral batch.

Table S10. Color change and staining of washing (1 and 5 cycles), rubbing (wet and dry) and UV light (4 h) fastness of the CO and PES fabrics dyed with prodigiosin alkaline batch without and with surfactant; dyed with neutral batch without and with surfactant.

	Samples	Washing fastness														Rubbing fastness				Light fastness (4 h)
		Colour change		Staining												Colour change		Staining		
				CA		CO		PA		PES		PAC		WO						
		1 W	5 W	1 W	5 W	1 W	5 W	1 W	5 W	1 W	5 W	1 W	5 W	1 W	5 W	Dry	Wet	Dry	Wet	
Alkaline batch	CO	1	1	5	5	5	5	5	5	5	5	5	5	5	5	5	5	5	5	5
	CO + Tween 80 (0.44 mM)	1	1	5	5	5	5	5	5	4/5	5	5	5	5	5	5	5	5	5	4
	PES	5	4	5	5	5	5	5	5	4/5	5	5	5	5	5	5	5	4	5	5
	PES + Tween 80 (0.44 mM)	4	4	4	5	4/5	5	4	5	3/4	5	3/4	5	4/5	5	5	5	5	5	4
Neutral batch	CO	4	3	3	5	4/5	5	3	5	2/3	4	4	5	3/4	5	4	4	4/5	3/4	3
	CO + Tween 80 (0.44 mM)	4	3	3	5	4/5	5	3	5	2/3	4	4	5	3/4	5	4	4	4/5	3/4	3
	CO+TA	3	3	4	5	4/5	5	3/4	5	3	4/5	4/5	5	4	5	-	-	-	-	3
	CO+Ch	4	4	4/5	5	4/5	5	4	5	3/4	4/5	4/5	5	4/5	5	-	-	-	-	2
	PES	4	3	5	5	5	5	5	5	4/5	5	5	5	5	5	5	5	3	4	3
	PES + Tween 80 (0.44 mM)	3	3	4/5	5	5	5	4/5	5	4	4/5	4/5	5	4/5	5	5	5	3	4	3

- *Application Biopigment in Conventional Printing: A Comparative Study of Commercial Thickener and Biopolymers*

Table S11. Color coordinates of CO and PES fabrics printed with prodigiosin using alkaline/neutral batches and thickened with Gilaba FF, sodium alginate or cellulose acetate.

				**CO Fabric**				**PES Fabric**			
				**L***	**a***	**b***	**Sample**	**L***	**a***	**b***	**Sample**
**Alkaline batch**	**Gilaba FF**	Control		70.63	14.42	-9.69	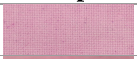	71.30	11.35	-8.82	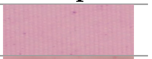
		Washing fastness	1 W	72.34	11.29	-4.89	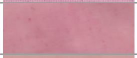	73.91	8.27	-3.13	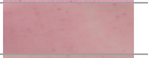
			5 W	77.04	7.31	0.15	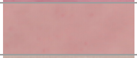	77.85	5.68	-0.50	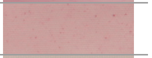
	**Sodium alginate**	Control		82.73	3.07	-0.40	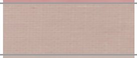	83.92	2.22	-1.43	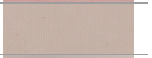
		Washing fastness	1 W	85.01	0.16	-0.26	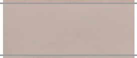	84.14	0.04	-1.07	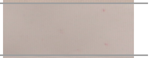
			5 W	84.45	0.08	-0.62	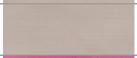	84.57	-0.02	-1.29	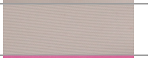
	**Cellulose acetate**	Control		58.01	21.80	-13.97	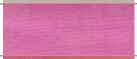	58.78	24.60	-11.31	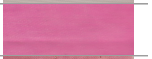
		Washing fastness	1 W	61.44	20.32	3.01	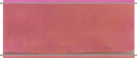	66.40	16.63	5.77	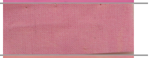
			5 W	62.38	19.38	0.72	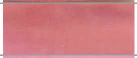	67.90	17.08	2.96	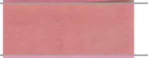
**Neutral batch**	**Gilaba FF**	Control		75.34	17.46	-7.53	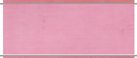	74.63	13.26	-6.60	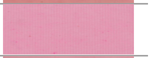
		Washing fastness	1 W	77.14	14.36	-3.97	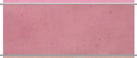	73.06	8.37	-2.15	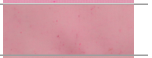
			5 W	79.88	6.01	0.73	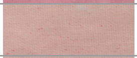	78.93	6.33	-2.29	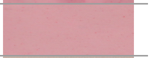
	**Sodium alginate**	Control		81.54	4.79	-0.77	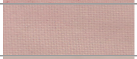	85.13	1.73	-1.16	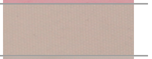
		Washing fastness	1 W	81.73	0.98	-0.75	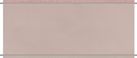	85.15	-0.04	-1.05	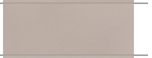
			5 W	83.98	0.19	-0.63	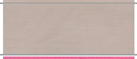	84.91	-0.11	-1.23	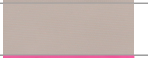
	**Cellulose acetate**	Control		64.67	28.58	-8.07	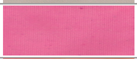	63.08	32.70	-13.68	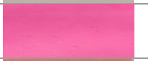
		Washing fastness	1 W	68.88	12.80	8.82	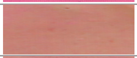	73.98	8.24	8.60	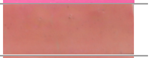
			5 W	75.57	13.76	4.71	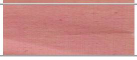	69.26	16.07	5.12	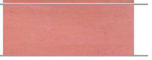

Table S12. Color change and staining of washing (1 and 5 cycles), rubbing (wet and dry) and UV light (4 h) fastness of the CO and PES fabrics printed with prodigiosin using alkaline/neutral batches and thickened with Gilaba FF, sodium alginate or cellulose acetate.

Samples			Washing fastness														Rubbing fastness				Light fastness (4 h)
			Colour change		Staining												Colour change		Staining		
					CA		CO		PA		PES		PAC		WO						
			1 W	5 W	1 W	5 W	1 W	5 W	1 W	5 W	1 W	5 W	1 W	5 W	1 W	5 W	Dry	Wet	Dry	Wet	
Gilaba FF	Neutral batch	CO	5	3	4/5	5	4/5	5	3	4/5	5	5	5	5	5	5	5	5	5	4/5	2
		PES	5	4	4/5	5	5	5	4/5	5	5	5	5	5	5	5	5	5	5	4/5	3
	Alkaline batch	CO	4	3	4/5	5	5	5	4/5	4/5	5	5	5	5	5	5	5	5	4/5	4	2
		PES	3	2	4/5	5	5	5	4	5	5	5	5	5	5	5	5	4	4/5	4	3
Sodium alginate	Neutral batch	CO	2	1	4	5	4/5	5	3/4	5	4	5	4/5	5	4	5	4	3	3/4	3/4	2
		PES	1	1	4/5	5	5	5	4	5	4/5	5	5	5	5	5	4	4	4/5	5	2
	Alkaline batch	CO	1	1	4/5	5	4/5	5	4	5	5	5	5	5	5	5	5	5	4/5	4/5	2
		PES	1	1	5	5	5	5	4/5	5	5	5	5	5	5	5	5	4	5	4/5	3
Cellulose acetate	Neutral batch	CO	3	2	5	5	4/5	5	2/3	4	4/5	5	4	5	3/4	5	5	4	5	5	2
		PES	3	3	5	5	5	5	5	4/5	5	5	5	5	5	5	5	4	5	5	3
	Alkaline batch	CO	2	3	5	5	5	5	5	5	5	5	5	5	5	5	4	4	4/5	4/5	2
		PES	3	3	5	5	5	5	5	5	5	5	5	5	5	5	5	3	5	4/5	3

References

1. Liu, J.; Yang, M.; Tan, J.; Yin, Y.; Yang, Y.; Wang, C. pH-responsive discoloration silk fibroin films based on prodigiosin from microbial fermentation. *Dyes and Pigments* **2022**, *198*, doi:10.1016/j.dyepig.2021.109994.
2. Ribeiro, A.I.; Vieira, B.; Alves, C.; Silva, B.; Pinto, E.; Cerqueira, F.; Silva, R.; Remião, F.; Shvalya, V.; Cvelbar, U.; et al. Halochromic Silk Fabric as a Reversible pH-Sensor Based on a Novel 2-Aminoimidazole Azo Dye. *Polymers* **2023**, *15*, doi:10.3390/polym15071730.
3. Venil, C.K.; Dufossé, L.; Velmurugan, P.; Malathi, M.; Lakshmanaperumalsamy, P. Extraction and Application of Pigment from Serratia marcescens SB08, an Insect Enteric Gut Bacterium, for Textile Dyeing. *Textiles* **2021**, *1*, 21-36, doi:10.3390/textiles1010003.
4. Ren, Y.; Gong, J.; Fu, R.; Zhang, J.; Fang, K.; Liu, X. Antibacterial dyeing of silk with prodigiosins suspention produced by liquid fermentation. *J. Cleaner Prod.* **2018**, *201*, 648-656, doi:10.1016/j.jclepro.2018.08.098.
5. Metwally, R.A.; El Sikaily, A.; El-Sersy, N.A.; Ghozlan, H.A.; Sabry, S.A. Antimicrobial activity of textile fabrics dyed with prodigiosin pigment extracted from marine Serratia rubidaea RAM_Alex bacteria. *Egyptian Journal of Aquatic Research* **2021**, *47*, 301-305, doi:10.1016/j.ejar.2021.05.004.
6. Alihosseini, F.; Ju, K.S.; Lango, J.; Hammock, B.D.; Sun, G. Antibacterial Colorants: Characterization of Prodiginines and Their Applications on Textile Materials. *Biotechnol. Prog.* **2008**, *24*, 742-747, doi:10.1021/bp070481r.
